# Trait-Structured Functional Reorganization Beneath Concordant Taxonomic and Functional Diversity in China’s Wintering Waterbirds

**DOI:** 10.3390/ani16203167

**Published:** 2026-10-09

**Authors:** Shi Dong, Hongtao Zhong, Lingfeng Huang

**Affiliations:** 1 Key Laboratory of the Ministry of Education for Coastal and Wetland Ecosystems, College of the Environment and Ecology, Xiamen University, Xiamen 361102, China; dongshi0101@gmail.com (S.D.); zhonght@xmu.edu.cn (H.Z.); 2 National Observation and Research Station for the Taiwan Strait Marine Ecosystem, Xiamen University, Xiamen 361005, China

**Keywords:** wintering waterbirds, taxonomic diversity, functional diversity, land use, waterbird conservation

## Abstract

Land-use change affects biodiversity by altering both species composition and ecological functions. Taxonomic and functional diversity provide complementary perspectives, but their relationship can vary depending on how species traits are reorganized within communities. Here, we investigated nationwide patterns of wintering waterbird diversity in China by integrating bird observations (2015–2024), land-use information, and multidimensional functional traits. We examined spatial patterns of taxonomic and functional diversity, their relationship across landscapes, and whether species-specific associations with land-use gradients were structured by functional traits. Taxonomic and functional diversity showed strong spatial concordance and broadly similar associations with major land-use gradients. However, observed functional diversity was generally lower than expected under randomized species–trait correspondence, indicating non-random trait organization relative to the specified null model. Trait associations differed among land-use gradients, but only the agricultural-landscape gradient showed FDR-supported improvement in prediction for held-out species. These results show that strong TD–FD concordance can coexist with non-random trait organization and does not make the two biodiversity dimensions interchangeable. Joint consideration of species richness and functional traits can therefore provide a more complete basis for conserving wintering waterbird diversity in human-modified landscapes.

## Simple Summary

Land-use transformation can alter both the number of bird species in a community and the ecological functions those species represent. Using nationwide observations of wintering waterbirds across China from 2015 to 2024, we examined whether taxonomic diversity and functional diversity showed similar spatial patterns and whether similar diversity levels could conceal differences in functional organization. We found that the two dimensions were strongly synchronized across China and showed broadly similar associations with major land-use gradients. However, waterbird communities consistently occupied a smaller range of functional trait space than expected if traits were randomly reassigned among species, indicating that strong diversity synchrony can coexist with non-random functional organization. We also found that species with different functional traits differed in their associations with different landscape gradients. These findings show that similar patterns in species and functional diversity do not necessarily mean that functional information is redundant, and that examining the organization of functional traits provides a more complete picture of how waterbird communities are structured across changing landscapes.

## 1. Introduction

Land-use transformation is a primary driver of global biodiversity reorganization, altering not only the species richness within assemblages but also the ecological strategies and functional attributes they represent [1,2]. Taxonomic diversity (TD) captures variation at the species level, whereas functional diversity (FD) describes the breadth of functional traits and ecological strategies represented within an assemblage, as determined by the traits of the constituent species and their distribution in multidimensional trait space [3]. These two dimensions are complementary yet intrinsically linked. As species richness increases, assemblages generally occupy a larger portion of functional trait space [4,5], generating concordant changes in TD and FD. Conversely, when species loss, gain or turnover is functionally selective, shifts in functional space may deviate from expectations based on species numbers alone, leading to a decoupling of the two diversity dimensions [6,7]. Jointly examining TD and FD under land-use transformation can therefore distinguish parallel expansion or contraction of taxonomic and functional diversity from disproportionate functional reorganization.

Studies of birds at global and regional scales further indicate that landscape composition and land-use heterogeneity shape both taxonomic and functional diversity, whereas agricultural intensification, urbanization and habitat conversion can selectively filter species with different ecological strategies, causing functional change to diverge from species-richness-based expectations [2,6,8]. The relationship between TD and FD is therefore likely to be strongly scale- and context-dependent [9], underscoring the need for joint assessments across broad landscape gradients. China provides a particularly informative system for addressing this question. Rapid urbanization and extensive land-use transformation have created highly heterogeneous landscapes in which natural wetlands, agricultural areas and impervious surfaces are juxtaposed across much of the country [10]. China also lies at the core of the East Asian–Australasian Flyway and provides critical habitat for large numbers of migratory and wintering waterbirds [11,12]. These species span pronounced gradients in body size, bill morphology, mobility, foraging strategy and aquatic habitat dependence, such that similar changes in species diversity may have markedly different functional consequences [13,14]. This combination of strong land-use gradients, high conservation importance and marked ecological differentiation makes China’s wintering waterbirds a powerful system for examining how taxonomic and functional diversity are jointly organized at a continental scale.

Previous research on Chinese wintering waterbirds has revealed links between environmental variation and assemblage functional structure across multiple spatial scales. Regional comparisons have identified marked geographical differences in the functional structure [13], whereas studies of individual wetlands and regional wetland networks have shown that water depth, hydrological conditions, aquatic vegetation and wetland restoration are associated with variation in functional diversity and community assembly [15,16]. Landscape composition surrounding lakes has likewise been related to taxonomic, functional and phylogenetic diversity [17], while long-term landscape transformation along the Yellow Sea coast has coincided with systematic shifts in the distributions of body-size and dietary traits within particular waterbird groups [18]. Together, these studies demonstrate that the composition and functional structure of Chinese wintering waterbird assemblages are closely linked to environmental variation operating from local habitat conditions to regional landscape configuration. However, most previous work has focused on individual wetlands, regional wetland networks, particular ecological groups or single biodiversity dimensions, leaving unresolved how taxonomic and functional diversity are jointly organized across national-scale land-use gradients. In particular, it remains unclear to what extent the large-scale relationship between TD and FD arises from structural coupling between assemblage diversity and represented functional trait space, and whether species-specific associations with land-use gradients retain systematic functional-trait structure beneath this assemblage-level relationship.

Here, we address these questions by linking assemblage-level diversity patterns with species-level functional-trait structure across national-scale land-use gradients in China. Specifically, we ask three questions: (1) What spatial patterns do TD and FD exhibit across China’s wintering waterbird assemblages, and how are these patterns associated with major land-use gradients? (2) What is the national-scale relationship between TD and FD, and to what extent can this relationship be explained by the structural coupling between assemblage diversity and represented functional trait space? (3) Do species differ systematically in their associations with major land-use gradients, and can these interspecific differences be explained by functional traits? By connecting these two levels of ecological organization, our study seeks to clarify how waterbird assemblages are reorganized across human-modified landscapes and to identify ecological strategies that differ systematically in their associations with landscape transformation, thereby providing a stronger basis for trait-informed waterbird conservation.

## 2. Materials and Methods

### 2.1. Study System, Species Pool and Checklist Data

This study analyzed 296 waterbird species recorded in China [19]. Waterbirds were defined according to the Ramsar waterbird concept. The complete species list, taxonomic classification, and IUCN Red List status are provided in the Supplementary Information (Supplementary Table S1). Prior to integrating checklist and trait data, species names were harmonized to eBird taxonomy v2025. Complete checklists were obtained from the eBird Basic Dataset and Sampling Event Data (EBD_relMar-2026; eBird Basic Dataset, 2026). We retained observations collected during the period 2015–2024 under the eBird Stationary or Traveling protocols, following established recommendations for ecological inference from semi-structured eBird data [20]. Checklists were required to report all species and to have survey durations > 0 and ≤300 min. Traveling surveys were restricted to ≤10 km where distance information was available. Shared checklists bearing the same group identifier were represented by a single record, and missing observer counts were set to one.

**Figure 1:**
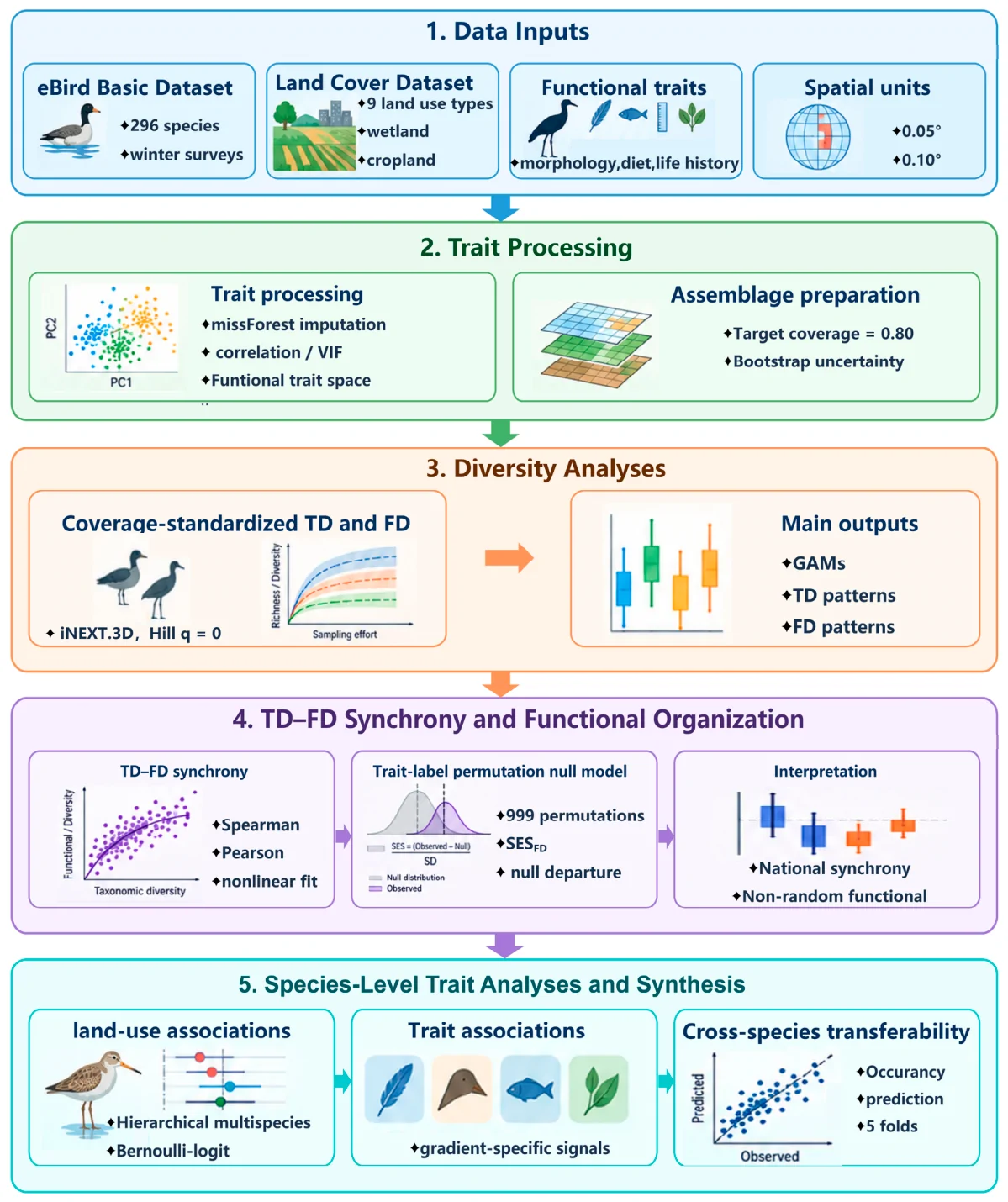
Conceptual workflow of the analytical framework. The workflow summarizes the main analytical steps of this study. First, four data sources were assembled: eBird winter checklist data, the China land-cover dataset, the functional-trait database, and spatial sampling units. Second, trait processing and assemblage preparation were conducted, including trait imputation, screening for redundancy and multicollinearity, dimensionality reduction, and construction of coverage-standardized assemblages. Third, assemblage-level taxonomic diversity (TD) and functional diversity (FD) were estimated and related to land-use gradients. Fourth, TD–FD synchrony was quantified and compared against a trait-label permutation null model to assess non-random functional organization. Fifth, species-level land-use associations were modeled and linked to the seven functional dimensions, including evaluation of cross-species transferability.

Analyses were restricted to the wintering period of November–February. Observations from November and December were assigned to the following calendar winter year, yielding winter seasons from 2016 to 2024. The winters of 2016–2019 were classified as period P1, and those of 2020–2024 as period P2. The filtered dataset contained 34,054 complete checklists, from which we constructed a checklist × species incidence matrix; for complete checklists, target species not reported were treated as non-detections. Coverage-standardized assemblage analyses used 33,390 checklists meeting the final sampling criteria, whereas species-level land-use models used 33,262 checklists with valid land-use support and ≤10 observers. Of the 296 species, 176 had sufficient direct checklist support for the primary species-level analyses; the remaining 120 species, including 116 all-zero species and four predominantly marine species lacking valid terrestrial CLCD support, were retained in the regional species pool and assemblage-level analyses and were included only in model-assisted sensitivity estimates. Additional sensitivity analyses assessed the representativeness and robustness of the species-level results with respect to functional-trait coverage, observational support, species detectability, and spatial clustering (Supplementary Methods S9–S12). In addition, across all 0.05° grid cells within the potential waterbird habitat domain, we summed the predicted encounter probabilities of all 296 species under standardized survey conditions to derive a standardized expected encounter richness index for describing nationwide spatial patterns. An overview of the analytical workflow is provided in Figure 1.

### 2.2. Land-Use Gradients and Functional Traits

Land-cover composition was derived from the annual China Land-Cover Dataset (CLCD; [21]), transformed from the original Albers projection to WGS84, and aggregated to a 0.05° geographic grid. Each annual layer contained proportional cover of cropland, forest, shrubland, grassland, water, snow/ice, barren land, impervious surface and wetland. Annual layers from 2015 to 2024 were used; environmental conditions for P1 and P2 were represented by means for the periods 2015–2019 and 2020–2024, respectively. Boundary grid cells were clipped to the national study domain, and retained land-cover proportions were renormalized to sum to 100%.

The potential waterbird habitat domain comprised 0.05° grid cells in which water plus wetland cover exceeded zero in at least one year during the period 2015–2024, yielding 181,054 candidate cells. Assemblage-level models characterized broad gradients in aquatic-resource availability, open-water cover, cropland, impervious cover and landscape-edge structure. All continuous landscape predictors were standardized before modeling. Because wetland cover was zero in 99.80% of checklist units relevant to the agricultural contrast, the agricultural predictor was interpreted as an agricultural-landscape gradient rather than as a measure of direct wetland-to-cropland conversion.

At the species level, the same broad landscape dimensions were represented using compositional log-ratio contrasts to reduce dependence arising from the constant-sum structure of land-cover proportions [22]. Let *W*, *H*, *I*, *F*, *G* and *C* denote the proportional cover of water, wetland, impervious surface, forest, grassland and cropland, respectively. The three focal contrasts were:

$$L_{water}=\log\left(\frac{W+H+0.01}{100-W-H+0.01}\right)$$

$$L_{impervious}=\log\left(\frac{I+0.01}{F+G+H+0.01}\right)$$

$$L_{cropland}=\log\left(\frac{C+0.01}{H+0.01}\right)$$

These parameterizations were used to estimate species-specific associations with aquatic resources, impervious surfaces and agricultural landscapes, respectively. These land-use variables were therefore treated as broad landscape-level proxies rather than direct measures of local habitat conditions or specific ecological mechanisms.

Functional diversity and species-level trait analyses were based on the same seven-dimensional functional trait space constructed for the 296-species regional pool. Morphological traits were obtained primarily from AVONET [14], life-history traits from the Amniote Database [23], and dietary composition from EltonTraits 1.0 [24]. Missing values among retained traits were imputed using missForest [25], and candidate variables were screened for redundancy and multicollinearity following established ecological-modeling practice [26]. The candidate trait variables were organized into five ecological modules: body size, trophic morphology, locomotion and dispersal capacity, life-history strategy, and resource use. Strongly correlated variables (|r| > 0.7) and variables with high multicollinearity (VIF ≥ 5) were screened to reduce redundancy, while PCA was used within correlated trait groups to summarize major axes of variation. Body size was summarized by the first principal component of log-transformed body mass, body length and wing length, whereas beak morphology was represented by the first two principal components of log-transformed bill length, bill width and bill depth. The final functional space comprised body size (Size_PC1), beak morphology (Beak_PC1 and Beak_PC2), hand-wing index (HWI), log-transformed tarsus length, log-transformed clutch size and diet composition (Diet_PC1). All seven dimensions were standardized to zero mean and unit variance across the 296-species pool. Because the retained axes were continuous and standardized, Euclidean distance was used to quantify multivariate trait dissimilarity without differences in measurement scale dominating the analysis.

Under the retained PCA orientation, higher Size_PC1 scores corresponded to generally smaller-bodied species; Beak_PC1 primarily represented overall bill size, whereas Beak_PC2 contrasted relatively long and narrow bills with shorter, broader or deeper bill morphologies. Detailed trait sources, preprocessing, missing-data treatment, variable screening, PCA loadings and sensitivity analyses to alternative trait-space dimensionalities are provided in the Supplementary Methods and in Tables S3–S6. The same standardized seven-dimensional matrix was used for both assemblage-level functional-diversity estimation and species-level trait analyses.

### 2.3. Taxonomic and Functional Diversity

Taxonomic and functional diversity were standardized for sampling completeness using iNEXT.3D v1.0.12 and coverage-based rarefaction/extrapolation [27]. Incidence data were analyzed using estimate3D() with datatype = “incidence_raw” and coverage-based standardization. The primary analysis used a sample-coverage target of 0.80, with coverage = 0.90 evaluated as a sensitivity analysis. Hill-number orders *q* = 0 and *q* = 1 were estimated, with *q* = 0 retained for the principal analyses reported here.

Spatial units were required to contain at least 10 checklists, observations from at least three winter months and three winter years, as well as detections of at least five target species. Extrapolation to the target coverage was additionally constrained such that:

*m_T_* ≤ 2*T*

where *T* is the observed number of incidence sampling units and *m_T_* is the number of sampling units required to reach the target coverage. Coverage thresholds were not relaxed for undersampled grid cells.

Taxonomic diversity (TD) was estimated as coverage-standardized species richness. Functional diversity (FD) was calculated from Euclidean pairwise distances among species in the standardized seven-dimensional trait space. Distances were normalized by the maximum pairwise distance across the complete 296-species pool, and FD was estimated using the area-under-the-curve formulation implemented in iNEXT.3D (diversity = “FD”, FDtype = “AUC”, FDcut_number = 50; [27]).

TD and FD were estimated preferentially at 0.05° resolution. Where a 0.05° grid cell did not satisfy the predefined sampling-completeness criteria, diversity was evaluated at the corresponding 0.10° support according to the predefined spatial-support rules. The final assemblage analysis contained 456 coverage-standardized spatial units, comprising 379 units at 0.05° resolution and 77 units at 0.10° resolution. Diversity estimates incorporated bootstrap uncertainty, which was subsequently propagated through 200 repeated response draws in the assemblage-level models.

### 2.4. National Diversity Patterns and Land-Use Associations

Associations between land use and TD or FD were evaluated separately using generalized additive models (GAMs; [28]). For spatial unit i, the response was:

*y_g_* = log(*D_g_*)

where *D_g_* denotes the coverage-standardized TD or FD estimate.

To distinguish land-use information from broad geographic structure, we compared a spatial baseline model with a full land-use model. Writing R for the spatial-resolution term, the two models were:

*M*_0_: *y*∼*R* + *s*(*lon*, *lat*)

*M*_1_: *y*∼*R* + *s*(*lon*, *lat*) + *s*(*z_aq_*) + *z_imp_* + *z_crop_* + *z_open_* + *z_edge_*

The resolution term distinguished between 0.05° and 0.10° support units. The broad geographic structure was represented by a two-dimensional thin-plate spline of longitude and latitude (k = 100). The aquatic-resource gradient was fitted using a cubic regression spline (k = 5), whereas the remaining standardized land-use predictors entered as linear terms.

Models were fitted in mgcv using restricted maximum likelihood (method = “REML”) with term selection enabled (select = TRUE). Spatial units were weighted according to the relative uncertainty of their diversity estimates:

$$w_{g}=\frac{1}{{\left(SE_{g}/D_{g}\right)}^{2}}$$

Predictive performance was evaluated using five-fold spatial cross-validation to reduce optimistic error estimates arising from spatial dependence [29]. For each diversity dimension, geographically transferable information attributable to land use was quantified as the reduction in out-of-fold root-mean-square error (RMSE) of the full model relative to the spatial baseline. For standardized linear predictors, effect sizes were expressed as percentage differences in expected diversity associated with a one-standard-deviation increase in the predictor. Residual spatial autocorrelation was evaluated using Moran’s I with 999 permutations [30]. Robustness was further examined across individual spatial folds, the alternative coverage target, the 200 repeated response draws, and a spatially balanced resampling analysis in which one assemblage unit was sampled from each occupied 0.25° grid block across 200 iterations (Supplementary Table S17).

### 2.5. TD–FD Synchrony and Its Functional Basis

National-scale synchrony between TD and FD was quantified using complementary rank-based, linear and nonlinear measures. We calculated Spearman’s rank correlation between coverage-standardized TD and FD and Pearson’s correlation after log-transforming both diversity dimensions. We additionally fitted a prespecified nonlinear relationship:

log(*FD_i_*) = *β*_0_ + *s*(log(*TD_i_*)) + *ε_i_*

using a cubic regression spline (bs = “cr”, k = 5) fitted in mgcv by restricted maximum likelihood [28]. The proportion of deviance explained quantified the extent to which broad variation in FD tracked variation in TD.

To evaluate the functional basis of the observed synchrony, we used a trait-label permutation null model that preserved the complete checklist × species incidence structure, species composition within spatial units, species occurrence frequencies, the seven-dimensional trait distribution and covariance structure, and the coverage-standardization procedure. The only randomized component was the correspondence between species identity and functional strategy: in each permutation, the complete seven-dimensional trait vector was reassigned among species as a single unit. For each of 999 permutations, FD was recalculated for the same 456 spatial units using the identical iNEXT.3D AUC definition and coverage target applied to the observed data. Spearman correlation, log-scale Pearson correlation and nonlinear deviance explained were then recalculated for each null realization and compared with their observed values. Randomization used a fixed seed and was implemented using checkpointed, reproducible simulation.

For each spatial unit i, richness- and incidence-constrained functional departure was summarized as:

$$SES_{FD,g}=\frac{FD_{\mathrm{obs},g}-\mu \left(FD_{\mathrm{null},g}\right)}{\sigma \left(FD_{\mathrm{null},g}\right)}$$

where *FD*_obs,*g*_ is the observed functional diversity and the null mean and standard deviation were calculated across the 999 trait-label permutations. Values near zero indicate that observed FD is close to that expected under randomized species–trait correspondence given the observed assemblage and incidence structure, whereas positive or negative values indicate FD greater or lower than the corresponding null expectation, respectively. As a secondary diagnostic, spatial structure in SES*_FD_* was evaluated using the same predefined spatial folds and land-use predictor definitions as the primary assemblage models; this analysis was treated as supplementary rather than as a primary test of nationwide TD–FD synchrony.

### 2.6. Trait Structure Beneath Assemblage-Level Synchrony

To test whether strong assemblage-level TD–FD synchrony nevertheless coexisted with selective functional reorganization, species-specific land-use associations were estimated independently of functional traits using a hierarchical multispecies Bernoulli-logit model. Because the response was checklist-level detection/non-detection rather than abundance, species not reported on complete checklists were treated as Bernoulli non-detections. For checklist i and species s:

*Y_is_*∼*Bernoulli*(*p_is_*)

$$logit\left(p_{is}\right)=\theta_{s0}+\sum_{j=1}^{38}X_{ij}\theta_{sj}$$

where *X_ij_* denotes checklist-level survey, temporal, spatial or land-use predictor *j* and *θ_sj_* is the corresponding species-specific coefficient. To stabilize species-level estimates while retaining interspecific variation, coefficients were represented hierarchically as:

***θ****_s_* = ***θ****_community_* + ***θ****_family_*_(*s*)_ + ***θ****_order_*_(*s*)_ + ***ϵ****_s_*

where the terms represent community-, family-, order- and species-level components, respectively. Functional traits were not included in this model and therefore contributed no information to the estimation of species-specific land-use coefficients.

The model included 38 checklist-level predictors: log-transformed survey duration, traveling distance and number of observers; observer experience; survey protocol; year; sine and cosine terms representing seasonal timing; longitude and latitude; the three focal land-use log-ratios; and 25 Gaussian spatial radial-basis functions. All continuous predictors were standardized before model fitting.

The hierarchical occurrence model was implemented in PyTorch (version 2.10.0) [31] using weighted Bernoulli cross-entropy and the Adam optimizer [32], with a batch size of 1024. After functional traits had been removed from the first-stage model, training duration was re-estimated using five spatial preflight folds. The median selected duration was 25 epochs and was subsequently used for five independently initialized full-data fits with distinct random seeds.

Functional traits were introduced only after species-specific land-use coefficients had been estimated. For each focal land-use gradient g, the independently estimated species coefficient was modeled simultaneously against the seven standardized functional dimensions:

$$\beta_{s,g}=\alpha_{g}+\sum_{k=1}^{7}\gamma_{k,g}T_{s,k}+\varepsilon_{s,g}$$

The three land-use gradients × seven functional dimensions yielded 21 prespecified trait–coefficient relationships. Uncertainty in these relationships was evaluated using 1000 bootstrap replicates. In each replicate, one of the five independently fitted occurrence models was randomly selected, species were resampled with replacement, and the seven-trait model was refitted.

Cross-species transferability was evaluated using five species-holdout folds defined before examination of the land-use coefficients. The 176 direct-data species were stratified by ecological group and wetland-specificity class and assigned to folds of 35, 35, 35, 35 and 36 species. For each fold, the trait–coefficient model was fitted to the remaining four folds and used to predict the independently estimated land-use coefficients of held-out species. Predictions were compared with a no-trait baseline defined as the mean coefficient among training species.

Predictive improvement was quantified as:

Δ*RMSE* = *RMSE_baseline_* − *RMSE_trait_*

such that positive values indicate lower prediction error when functional traits were included. Overall uncertainty in ΔRMSE was quantified using 3000 joint bootstrap replicates that randomly selected one occurrence-model fit, resampled the complete set of out-of-fold species with replacement, and recalculated predictive improvement. The three gradient-level tests were corrected for multiple comparisons using the Benjamini–Hochberg false-discovery-rate procedure [33]. Additional guild comparisons and extended sensitivity analyses were treated as supplementary analyses.

## 3. Results

### 3.1. National Land-Use Gradients Are Associated with Concordant Taxonomic and Functional Diversity Patterns

**Figure 2:**
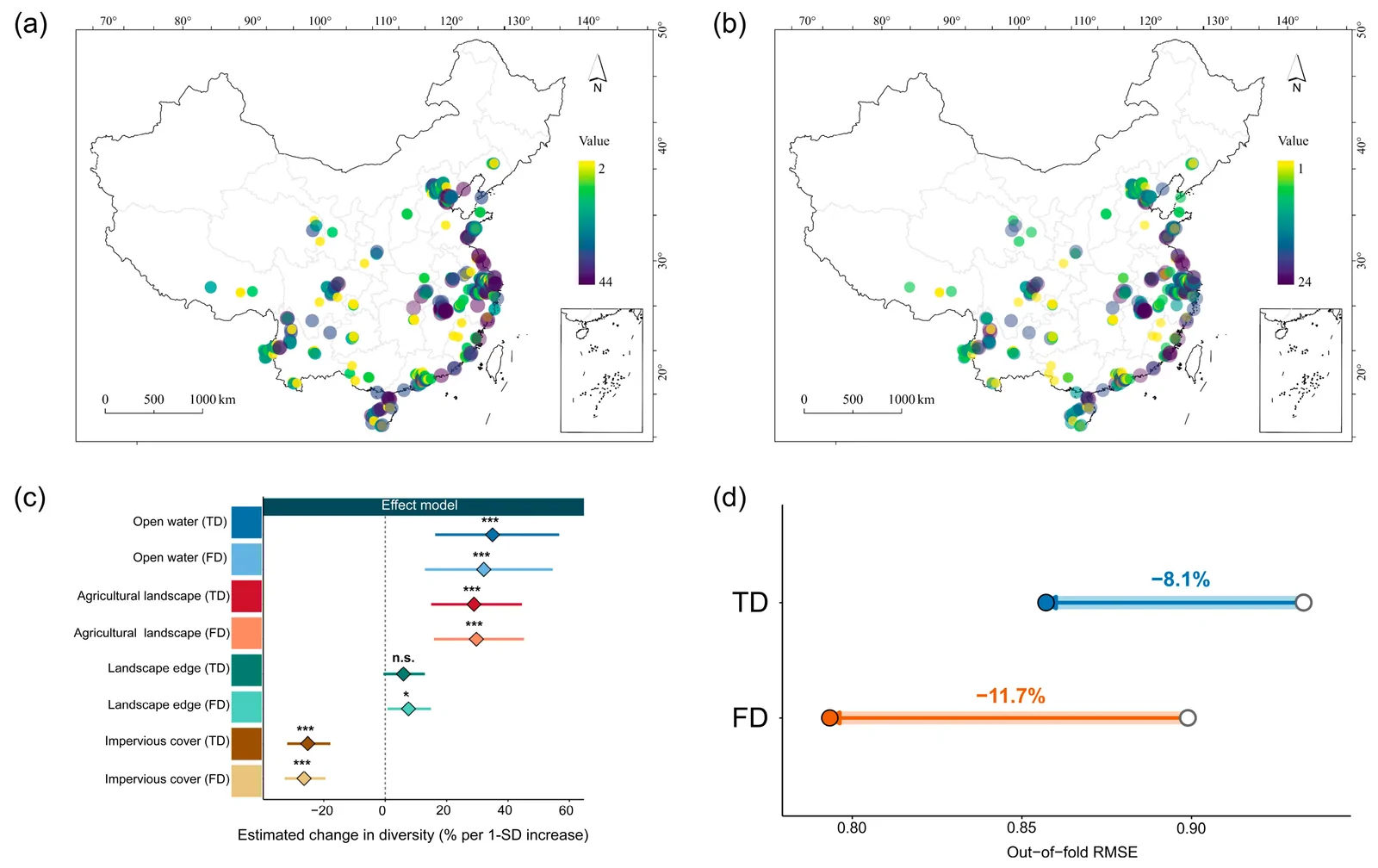
National patterns of taxonomic and functional diversity and their associations with land use. (**a**,**b**) Coverage-standardized taxonomic diversity (TD) and functional diversity (FD; Hill number *q* = 0, sample coverage = 0.80) across 456 spatial units meeting the sampling-completeness criteria. Point colors indicate estimated diversity values using panel-specific scales. (**c**) Estimated percentage changes in TD and FD associated with a one-standard-deviation increase in each of the four land-use gradients, based on spatially explicit generalized additive models. Points represent effect estimates, and horizontal lines indicate 95% confidence intervals. Significance levels are indicated as follows: * *p* < 0.05; *** *p* < 0.001; n.s., not significant. (**d**) Out-of-fold root-mean-square error (RMSE) of the geographic-structure-only baseline model (open circles) and the full land-use model (filled circles) under five-fold spatial cross-validation. Percentages indicate the relative changes in RMSE of the full model compared with the baseline model.

Taxonomic diversity (TD) and functional diversity (FD) showed broadly concordant spatial patterns across China, with regions of relatively high and low diversity largely overlapping between the two dimensions (Figure 2a,b). TD and FD also exhibited highly similar associations with the major land-use gradients. A one-standard-deviation increase in open-water availability was associated with 34.94% higher TD and 32.11% higher FD, whereas a one-standard-deviation increase in impervious cover was associated with 25.25% lower TD and 26.43% lower FD. Agricultural landscape intensity was positively associated with both dimensions, corresponding to 28.90% higher TD and 29.70% higher FD per standard deviation. Landscape edge showed a weaker and less consistent pattern: FD was 7.59% higher per standard deviation, whereas the corresponding TD estimate was more uncertain and its interval overlapped zero (Figure 2c).

Land-use variables also improved spatially out-of-sample prediction of both diversity dimensions. Relative to the spatial baseline models, their inclusion reduced out-of-fold prediction error by 8.14% for TD and 11.74% for FD (Figure 2d). Residual spatial autocorrelation was not detected after model fitting, and the directions of the estimated associations were broadly consistent across spatial folds and sensitivity analyses (Supplementary Figure S3; Supplementary Tables S8–S10 and S17).

### 3.2. Taxonomic and Functional Diversity Are Strongly Synchronized Across China

**Figure 3:**
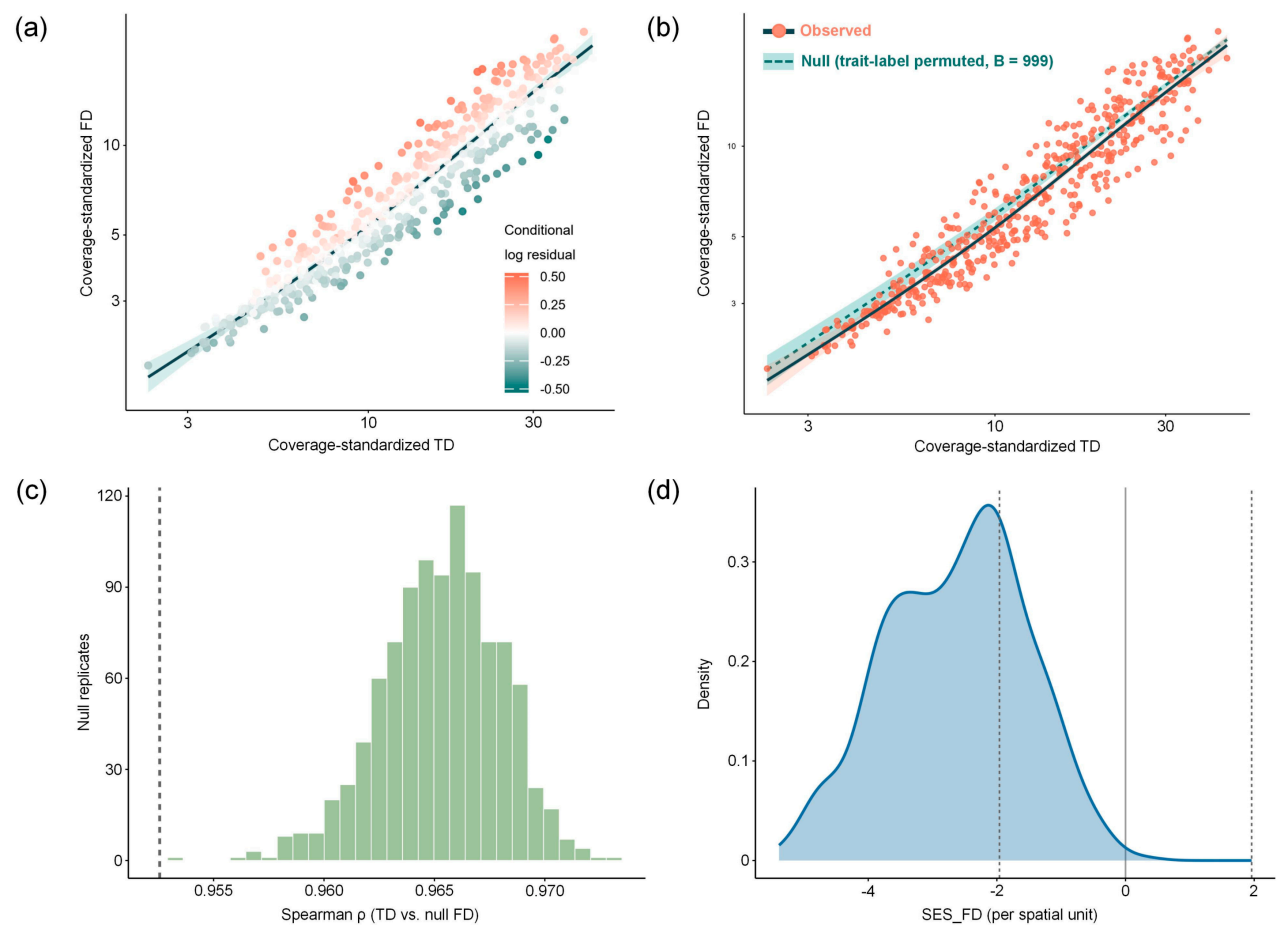
National TD–FD synchrony and departure from trait-label-permutation expectations. (**a**) Relationship between coverage-standardized taxonomic diversity (TD) and functional diversity (FD) across 456 spatial units on log–log scales, with the fitted nonlinear relationship and 95% confidence interval. Points are colored by their conditional log residuals from the fitted relationship. Spearman’s ρ = 0.953, and the nonlinear model explained 90.43% of the deviance in FD. (**b**) Observed TD–FD relationship (coral) compared with the trait-label-permutation null expectation (teal; 999 permutations). Trait-label-permutation null realizations were generated by permuting intact seven-dimensional trait vectors among species while preserving the observed incidence and assemblage structure; the dashed line and band show the null mean and 95% envelope. (**c**) Null distribution of Spearman’s ρ across the 999 permutations. The observed value (ρ = 0.953; dashed line) was lower than all null replicates (null mean = 0.965; empirical *p* = 0.001). (**d**) Distribution of unit-level standardized effect sizes for FD relative to the corresponding null expectation (SES*_FD_*; median = −2.54), where negative values indicate lower observed FD than expected under randomized species–trait correspondence.

Wintering waterbird assemblages showed strong national-scale concordance between TD and FD, with taxonomically more diverse assemblages generally occupying broader functional trait space (Figure 3a). Across the 456 spatial units, TD and FD were strongly correlated (Spearman’s ρ = 0.953; log-scale Pearson’s *r* = 0.950), and the prespecified nonlinear model explained 90.43% of the deviance in FD. The positive TD–FD relationship extended across the observed diversity gradient (Figure 3a).

Nevertheless, variation in FD remained among assemblages with similar TD. Assemblages with comparable taxonomic diversity did not invariably contain the same amount of functional trait space, producing measurable dispersion around the fitted national TD–FD relationship (Figure 3a). Additional diagnostics of conditional FD variation and taxonomic–functional mismatch showed little evidence for widespread extreme departures from the national relationship.

### 3.3. Observed TD–FD Synchrony Is Lower than Trait-Randomized Expectations

The observed TD–FD relationship differed consistently from the trait-randomized null expectation. Across much of the TD gradient, observed FD lay below the distribution expected under randomized species–trait correspondence while assemblage composition and incidence structure were retained (Figure 3b). The observed Spearman correlation was 0.9526, compared with a null mean of 0.9653 (95% null range: 0.9594–0.9701), and fell below all 999 null replicates (empirical *p* = 0.001; Figure 3c).

The same pattern was obtained using the two additional measures of TD–FD concordance. The observed log-scale Pearson correlation was 0.9498, compared with a null mean of 0.9630 (95% null range: 0.9570–0.9680), whereas nonlinear deviance explained was 90.43% in the observed data compared with a null mean of 92.85% (95% null range: 91.76–93.79%). Both observed values fell below all 999 corresponding null replicates (empirical *p* = 0.001; Supplementary Figures S4 and S5; Supplementary Table S11).

At the spatial-unit level, observed FD was also generally lower than its corresponding null expectation. Mean SES*_FD_* was −2.62 and median SES*_FD_* was −2.54, with 71.1% of the 456 spatial units falling outside their unit-specific 95% null envelopes (Figure 3d). The distribution was strongly shifted toward negative SES*_FD_* values, although the magnitude of departure varied among spatial units. In the secondary spatially validated analysis, adding the measured land-use gradients did not improve out-of-fold prediction of SES*_FD_* relative to the spatial baseline model (−1.83% improvement), and residual spatial autocorrelation remained significant (Moran’s *I* = 0.092, *p* < 0.001; Supplementary Figure S6; Supplementary Tables S12 and S13).

### 3.4. Functional Traits Are Associated with Species-Specific Land-Use Gradients

**Figure 4:**
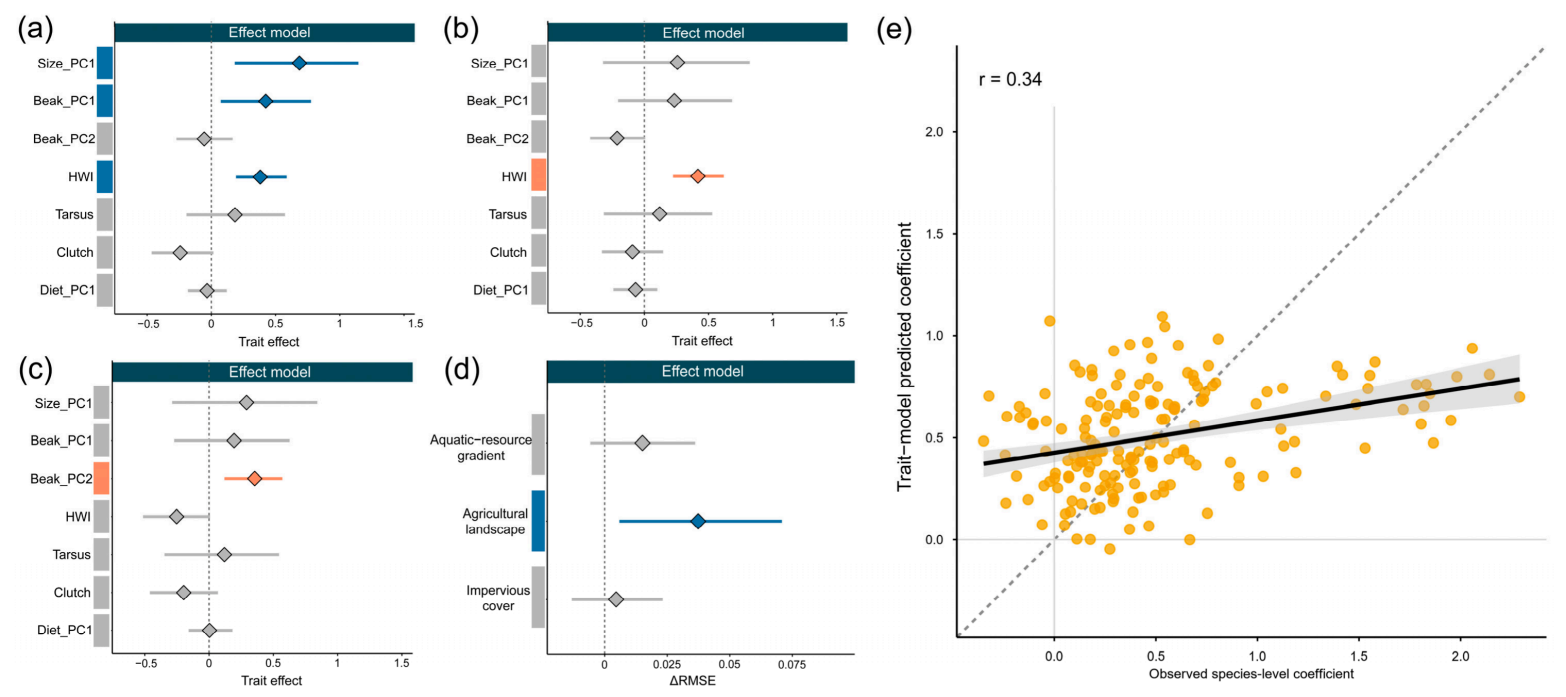
Trait associations with species-specific land-use coefficients and their cross-species transferability. Species-specific land-use coefficients were estimated without functional-trait information in the first-stage hierarchical multispecies model; functional traits were introduced only in the second-stage analyses shown here. (**a**–**c**) Standardized multivariate effects of the seven functional-trait axes on species-specific coefficients for the agricultural-landscape (**a**), aquatic-resource (**b**), and impervious-cover (**c**) gradients. Points show effect estimates and horizontal lines show 95% bootstrap confidence intervals. Blue highlights supported trait-level associations within the agricultural gradient, the only gradient showing false-discovery-rate (FDR)-supported species-holdout improvement; orange highlights supported trait-level associations within the aquatic and impervious gradients, for which multivariate holdout improvement was not FDR-supported; remaining relationships are shown in gray. (**d**) Improvement in species-holdout root-mean-square error (ΔRMSE) of the seven-trait model relative to a no-trait baseline. Positive values indicate improved prediction for held-out species; horizontal lines show 95% bootstrap confidence intervals. Holdout improvement was supported for the agricultural gradient (ΔRMSE = 0.0374, 95% CI = 0.0059–0.0708, FDR-adjusted *q* = 0.028), but not for the aquatic-resource (ΔRMSE = 0.0151, 95% CI = −0.0057–0.0362, *q* = 0.135) or impervious-cover gradients (ΔRMSE = 0.0046, 95% CI = −0.0132–0.0232, *q* = 0.316). (**e**) Observed agricultural-landscape coefficients plotted against out-of-fold trait-model predictions. The solid line shows the fitted relationship and the dashed line the 1:1 expectation (Pearson’s *r* = 0.34).

Species-specific land-use coefficients estimated independently of functional traits showed trait-associated variation that differed among the three land-use gradients. At the trait level, positive associations were supported for Size_PC1, Beak_PC1 and hand-wing index (HWI) along the agricultural-landscape gradient, for HWI along the aquatic-resource gradient, and for Beak_PC2 along the impervious-cover gradient (Figure 4a–c). However, the ability of the complete seven-trait model to generalize across species differed markedly among gradients (Figure 4d).

**Figure 5:**
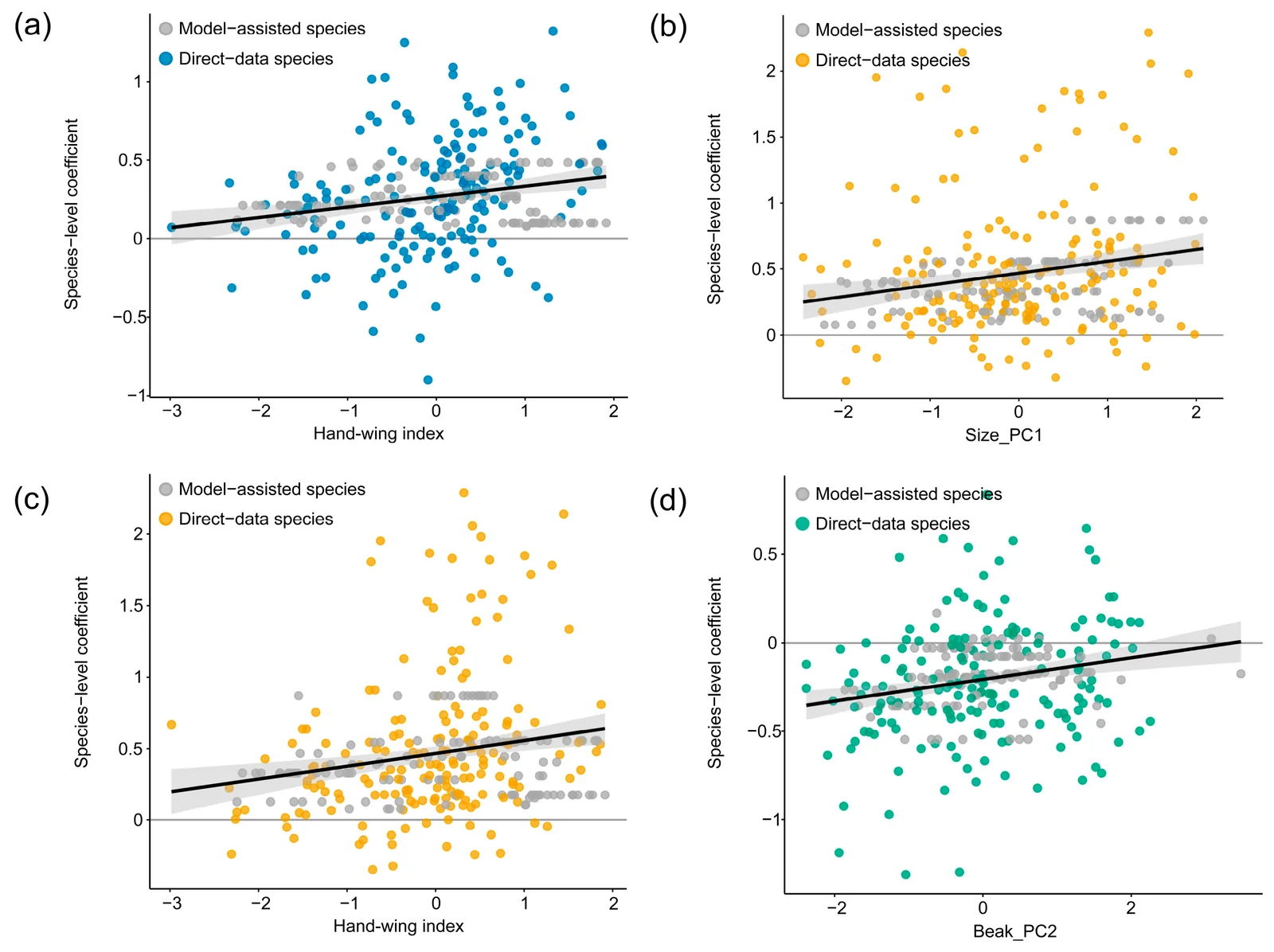
Representative functional-trait relationships underlying species-specific land-use associations. Species-specific land-use coefficients estimated independently of functional traits in the first-stage hierarchical multispecies model are plotted against standardized trait values for four relationships highlighted in the full trait-effect analysis (Figure 4a–c): (**a**) aquatic-resource coefficient versus hand-wing index (HWI); (**b**) agricultural-landscape coefficient versus Size_PC1, for which higher scores represent smaller-bodied species; (**c**) agricultural-landscape coefficient versus HWI; and (**d**) impervious-cover coefficient versus Beak_PC2. Colored points represent the 176 direct-data species used in the primary species-level analyses, whereas gray points represent the 120 model-assisted species included as a sensitivity set. Black lines show fitted relationships for the direct-data species, with shaded 95% confidence intervals.

Along the aquatic-resource gradient, HWI showed a positive association with species-specific aquatic coefficients (Figure 5a). At the multivariate level, however, functional traits did not significantly improve prediction for held-out species relative to the no-trait baseline (ΔRMSE = 0.0151, 95% bootstrap CI = −0.0057–0.0362; FDR-adjusted *q* = 0.135; Figure 4d).

The agricultural-landscape gradient showed the strongest and most transferable trait structure. Higher Size_PC1 scores, corresponding to smaller-bodied species, were associated with more positive agricultural coefficients (Figure 5b), while Beak_PC1 and HWI also showed positive trait-level associations (Figure 4a), with the HWI relationship illustrated in Figure 5c. The multivariate trait model reduced holdout RMSE by 0.0374 relative to the no-trait baseline (95% bootstrap CI = 0.0059–0.0708; FDR-adjusted *q* = 0.028; Figure 4d). Across the 176 direct-data species, held-out trait-based predictions were positively associated with the independently estimated agricultural coefficients (r = 0.34; Figure 4e).

Along the impervious-cover gradient, Beak_PC2 showed a supported positive association with species-specific impervious coefficients (Figure 5d). Nevertheless, this trait-level association did not translate into clear multivariate cross-species predictability: holdout improvement was small and uncertain (ΔRMSE = 0.0046, 95% bootstrap CI = −0.0132–0.0232; FDR-adjusted *q* = 0.316; Figure 4d). Additional trait-level estimates, bootstrap uncertainty and species-holdout diagnostics are provided in the Supplementary Information (Supplementary Tables S14–S16).

## 4. Discussion

### 4.1. Concordant Taxonomic and Functional Associations with Land Use

Across China, taxonomic diversity (TD) and functional diversity (FD) of wintering waterbird assemblages showed strikingly concordant spatial patterns. At the scale considered here, variation in species richness was generally accompanied by parallel expansion or contraction of the represented functional trait space, with little evidence for widespread assemblages that were taxonomically rich but functionally depauperate, or vice versa. Such concordance is partly expected because FD is constrained by species richness but also depends on where species are positioned within trait space [3]. Previous avian studies have documented similar concordant changes in TD and FD, including near-proportional temporal changes in British avian assemblages [4], whereas disproportionate functional losses have also been reported under land-use intensification [6], with functionally rare threatened shorebirds in China including the Spoon-billed Sandpiper (*Calidris pygmaea*) and Far Eastern Curlew (*Numenius madagascariensis*) [34]. At the broad spatial scale examined here, Chinese wintering waterbirds showed a comparable pattern of parallel taxonomic and functional variation.

Spatial scale may be central to this result [9]. Across broad geographical extents, species-rich assemblages generally encompass a wider range of functional strategies, strengthening the expected correspondence between TD and FD [5]. Global avian studies similarly show that landscape heterogeneity can be associated with increases in both taxonomic and functional diversity, whereas more direct or local habitat transformations can produce stronger restructuring of functional composition [2,8]. Comparable scale dependence has emerged from studies of Chinese waterbirds: landscape structure among wetlands can covary with multiple biodiversity dimensions, whereas within individual wetlands, water depth, vegetation structure and restoration processes can further reshape functional α- and β-diversity [15,16,17]. Thus, the strong nationwide TD–FD concordance observed here likely reflects, at least in part, the parallel scaling of assemblage diversity and represented trait space, while leaving open how that trait space is organized internally.

### 4.2. Non-Random Functional Organization Beneath TD–FD Synchrony

Strong TD–FD synchrony does not imply that random occupation of functional space expands passively with species number. Functional diversity reflects both the constraint imposed by species richness and the arrangement of species within trait space [3,5]. Assemblages with similar richness can therefore occupy markedly different amounts of functional space depending on species identity and trait configuration [35]. Our randomization analysis separated these two components. The nationwide TD–FD relationship had a strong taxonomic structural basis, yet observed assemblages did not fill the expanding trait space randomly; instead, they occupied less functional space than expected under the trait-label permutation null model, a pattern consistent with non-random functional organization relative to this null expectation. Species richness therefore constrains the broad range of FD, whereas the identities and trait combinations of co-occurring species determine how much of that space is actually realized. This concentration of species within a narrower trait space may increase redundancy among common ecological strategies while leaving distinctive strategies represented by fewer species [35]. In waterbird communities, this could concentrate functions such as predation, herbivory, seed dispersal and nutrient transport within a limited set of functional strategies [36].

This combination of strong synchrony and non-random organization has also been reported in other avian systems. Functional diversity can track species richness closely while remaining systematically lower than expected under randomized assemblage structure [4]. More broadly, the correspondence between TD and FD depends on the functional positions and distinctiveness of the species involved [4,35], and can vary substantially with spatial scale [9]. Continental-scale analyses further show that seasonality and productivity structure avian functional diversity and influence the balance between niche packing and functional expansion [37]. Together, these findings indicate that the strength of TD–FD coupling and the internal organization of trait space are related but distinct properties of assemblages.

For wintering waterbirds, the coexistence of strong coupling and compressed functional space may reflect processes operating at different scales. Broad richness gradients constrain the amount of trait space that assemblages can potentially occupy, whereas the realized filling of that space may additionally depend on shared wetland dependence, foraging ecology, hydrological conditions [38], as well as spatially structured species pools and phylogenetically conserved ecological traits [39]. Regional studies of waterbird assemblages have reported both pronounced functional clustering and communities closer to random functional expectations, suggesting that functional organization itself is scale- and context-dependent [40,41,42]. This interpretation is also consistent with our land-use analysis: the major landscape gradients robustly described broad spatial variation in TD and FD, but did not further predict departures of FD from its null expectation. Broad landscape context may therefore be more informative about overall diversity patterns, whereas finer-scale habitat structure and species-pool composition may play a greater role in determining how that diversity is organized in trait space. Importantly, the national-scale patterns detected here represent statistical variation among assemblages rather than the local ecological processes that generate functional structure within individual wetlands, such as competition or microhabitat selection. Targeted field validation at representative wetlands will therefore be important for determining whether the non-random functional organization detected here primarily reflects local assembly processes or broader-scale species sorting.

### 4.3. Different Land-Use Gradients Are Associated with Distinct Forms of Interspecific Functional Structure

Species–landscape associations showed clear landscape-specific functional structure. Agricultural landscapes exhibited the strongest multivariate trait signal: smaller-bodied species and species with higher hand-wing index (HWI) tended to show more positive associations, and this trait structure generalized across species. Along the aquatic gradient, interspecific differentiation was concentrated primarily along HWI. These asymmetries suggest that different land-use gradients are associated with different dimensions and strengths of functional differentiation rather than with a single, generalized response syndrome.

This landscape specificity was most evident along the agricultural gradient. Higher HWI is generally associated with greater flight efficiency, migration and dispersal capacity, a pattern consistent with the use of spatially dispersed and seasonally dynamic agricultural–wetland mosaics [43]. Rice fields, post-harvest croplands and seasonally inundated fields can provide temporary feeding and roosting opportunities, but their suitability depends strongly on farming stage, hydrological management and surrounding natural wetlands [38,44]. Agricultural wetlands can therefore constitute important components of broader wetland mosaics and, under suitable management, may serve as valuable surrogate foraging habitat for some waterbirds [45]. Their functional value, however, is strongly contingent on hydrological and agricultural management [44] and should not be assumed to be equivalent to that of natural wetlands across species or ecological functions. The relatively stable trait structure detected here is therefore better interpreted as differences in species–landscape associations among ecological strategies, rather than as evidence of a general benefit of agricultural expansion to waterbirds. The association with smaller body size was also statistically well supported, but its ecological basis is less straightforward; contrasting regional patterns suggest that it may depend on guild composition, agricultural system, hydrological regime and spatial scale [18].

Aquatic and impervious gradients revealed two different forms of functional structuring. Along the aquatic gradient, HWI was the most consistent trait signal, but broader multivariate trait structure did not generalize as strongly as it did for agriculture. One important limitation is that open-water availability is a coarse descriptor of aquatic habitat and does not capture water depth, hydroperiod, vegetation structure, food availability or wetland connectivity, all of which can strongly influence niche differentiation among waterbirds [38]. Similarly, impervious cover is a broad landscape-level proxy that can covary with multiple local habitat conditions, including water availability, disturbance regimes, shoreline structure and landscape connectivity [2,46]. These broad land-use gradients should therefore be interpreted as landscape-level proxies rather than direct measures of the finer-scale habitat conditions relevant to wintering waterbirds. Overall, different land-use gradients were associated with distinct patterns of interspecific functional differentiation rather than merely differing in the strength of a common response axis. By leveraging extensive citizen-science observations, our analysis captures broad species-level landscape associations across China, although species-specific detectability and observer bias remain limitations, particularly for rare and cryptic species; future work that explicitly models sampling effort and detectability could further resolve these associations at finer ecological scales.

### 4.4. Trait-Space Expansion and Functional Organization Jointly Shape TD–FD Coupling

TD–FD synchrony can be understood as the combined outcome of two components: the expansion of trait space associated with differences in species richness, and the non-random occupation of that space by real assemblages [5,37]. Because the relative importance of these components varies among systems, similar levels of taxonomic variation can correspond to very different levels of functional variation. Avian studies illustrate this clearly: scale-dependent increases in functional redundancy can weaken the correspondence between taxonomic and functional change [9], while seasonal redistribution of functionally distinctive species can either strengthen or decouple the two dimensions [37,47].

Chinese wintering waterbirds represent a case in which assemblages with higher TD generally occupied broader functional space, producing a strongly coupled nationwide TD–FD relationship, while observed FD remained systematically lower than expected under randomized species–trait correspondence, revealing substantial compression of realized functional space. Thus, the observed synchrony reflects both large-scale expansion of trait space with taxonomic diversity and non-random functional organization within assemblages. The former maintains strong nationwide coupling, whereas the latter generates persistent departures from random functional expectations. Similar TD–FD correlations may therefore conceal markedly different internal assemblage structures, depending on where species occur within functional space and how they are combined.

The ecological significance of this internal organization ultimately lies in how taxonomic change is translated into functional consequences. Loss of species sharing similar ecological roles can erode functional redundancy and make subsequent species losses translate more rapidly into functional decline, even when contemporary changes in TD or FD remain modest [2]. Functions represented by several ecologically similar species may therefore remain buffered when individual species decline. By contrast, functions associated with distinctive strategies may be more sensitive to species loss, potentially reducing the resilience of trophic regulation and nutrient redistribution in wetlands [36,48]. TD–FD coupling is therefore informative not simply because it describes whether two biodiversity dimensions covary, but because it reflects how strongly taxonomic change is converted into functional change and how functional redundancy modulates that conversion. For Chinese wintering waterbirds, the critical future question is not whether TD and FD will remain highly correlated per se, but whether equivalent amounts of taxonomic change will continue to produce comparable functional consequences. Once that correspondence shifts, TD–FD decoupling would no longer represent only disagreement between biodiversity metrics; it would indicate that assemblage reorganization is generating functional consequences disproportionate to changes in species number.

### 4.5. Conservation Implications for Chinese Wintering Waterbirds

From a conservation perspective, our results indicate that species richness alone may conceal reductions in functional representation. Jointly considering TD, FD, and trait-space organization can therefore help identify assemblages whose functional structure is not fully captured by richness, broaden the assessment of conservation and restoration outcomes, and detect shifts toward functional homogenization that richness-based monitoring would overlook. Because land-use gradients did not further predict departures of FD from its null expectation, assemblages with strongly negative SES*_FD_*, particularly those in which richness remains high but realized trait space is compressed, warrant particular attention. Regional SES*_FD_* screening showed that such below-null assemblages were concentrated in the Eastern Plains and in several provinces, including Jiangxi, Zhejiang, Shandong, and Jiangsu, which may serve as candidate areas for further regional conservation assessment.

Gradient-specific trait associations further suggest that management and restoration should remain dependent on habitat context, rather than assuming that broad land-use categories are functionally equivalent across waterbird species. Along the agricultural gradient, management should retain natural wetland patches, maintain suitable post-harvest winter flooding, and preserve hydrological connectivity, rather than treating farmland as a functional substitute for natural wetlands [38,44,45]. Along the impervious-cover gradient, TD and FD both declined markedly, but no stable multivariate trait signal emerged; restoration should therefore focus on overall wetland habitat quality, including shallow-water habitat, reduced shoreline hardening, and connectivity among wetland patches [38,46], rather than targeting a single functional guild. Future work integrating population trends, threat status, and migratory connectivity with SES*_FD_*-based screening could further refine conservation priorities and support more dynamic strategies for waterbirds across the full migratory cycle.

## 5. Conclusions

Our study provides a national-scale assessment of how taxonomic and functional diversity are jointly organized in China’s wintering waterbird assemblages across major land-use gradients. Taxonomic diversity and functional diversity were strongly synchronized across the country and showed broadly concordant associations with open water, agricultural landscapes and impervious cover. However, this close correspondence did not indicate random or functionally redundant assemblage structure. Observed communities consistently occupied less functional trait space than expected under a trait-label permutation null model, revealing widespread functional compression beneath the strong nationwide TD–FD relationship. Species-level analyses further showed that this functional organization was not uniform across landscape gradients. Agricultural landscapes exhibited the clearest and most transferable multivariate trait structure, whereas aquatic-resource and impervious-cover gradients showed more limited or trait-specific differentiation. Together, these findings demonstrate that the strength of TD–FD coupling and the internal organization of functional trait space capture related but distinct dimensions of biodiversity structure. Strong agreement between taxonomic and functional diversity can therefore conceal substantial non-random functional organization, emphasizing the value of examining not only how much functional diversity is present, but also how that diversity is assembled within trait space. More broadly, our results highlight the importance of integrating assemblage-level diversity patterns with species-level functional associations when evaluating biodiversity across human-modified landscapes. From a conservation perspective, future assessments should therefore consider not only species numbers but also the integrity and representation of functional strategies, with particular attention to assemblages showing strongly negative SES*_FD_* and to landscapes where particular trait strategies are comparatively underrepresented. Future research could extend these national-scale spatial patterns toward local ecological processes through targeted field validation at representative wetlands, while also incorporating temporal dynamics and additional factors. Species-level responses could be further evaluated using occupancy models that explicitly account for detectability, standardized monitoring data, or long-term population surveys. Future work could also incorporate temporal dynamics and additional factors, such as phylogenetic structure and dispersal history, to better resolve the processes underlying departures from the null expectation. Combining long-term population and community trajectories with migratory connectivity, climate change and other environmental drivers could reveal how taxonomic–functional coupling and trait-space organization are reshaped across seasons, years and migratory stages, providing a more complete understanding of the processes governing functional biodiversity change in migratory waterbirds.

## Data Availability

Waterbird occurrence data are available from the eBird Basic Dataset under its access conditions (March 2026 release). The CLCD v1.0.4 dataset is available from Zenodo at https://doi.org/10.5281/zenodo.15853565. Functional-trait data are available from AVONET, the Amniote Database and EltonTraits 1.0.

## Supplementary Materials

The following supporting information can be downloaded at: https://www.mdpi.com/article/10.3390/ani16203167/s1, Supplementary Methods S1–S12 provide detailed descriptions of data processing, functional-trait construction, diversity estimation, statistical modeling, and sensitivity analyses. Supplementary Results S1–S4 present additional robustness assessments and model-validation results. Figures S1–S8 illustrate nationwide diversity patterns, functional-trait relationships, TD–FD synchrony, null-model comparisons, and species-level land-use associations. Tables S1–S26 provide supporting information on species taxonomy, functional traits, diversity estimates, land-use effects, null-model results, cross-species prediction, sensitivity analyses, and regional functional vulnerability.
