# Supplementary material for: Trait-Structured Functional Reorganization Beneath Concordant Taxonomic and Functional Diversity in China’s Wintering Waterbirds
Source: Animals (Basel). 2026 Oct 9;16(20):3167. doi: 10.3390/ani16203167 (PMC13700750; doi:10.3390/ani16203167)
Supplement: Supplementary file 1 [file animals-16-03167-s001.zip › Supplementary_Information_revised_R2.pdf]

Supplementary Information

Supplementary Methods

S1. Species pool, taxonomy and checklist filtering

The regional pool comprised 296 waterbird species recorded in China under the Ramsar waterbird concept. Names were harmonized to eBird taxonomy v2025 before checklist, trait and ancillary data were joined. Complete eBird checklists from the March 2026 Basic Dataset and Sampling Event Data releases were restricted to China, November–February, 2015–2024, and the Stationary or Traveling protocols. We retained checklists with duration greater than 0 and no more than 300 min; Traveling checklists were limited to 10 km when distance was available. Shared checklists were represented once, missing observer counts were set to one, and unreported members of the target pool were treated as non-detections on complete checklists.

November and December observations were assigned to the following winter year, generating winters 2016–2024. The filtered incidence data contained 34,054 complete checklists. Coverage-standardized assemblage analyses used 33,390 checklists meeting the final spatial-support criteria. Species-level occurrence models used 33,262 checklists with valid land-use support and no more than 10 observers. Among the regional pool, 176 species had at least one eligible winter detection and valid terrestrial CLCD support and were therefore defined as direct-data species (D1) for the primary species-level analyses. All 296 species remained in the assemblage-level diversity analyses and national standardized encounter-richness product. Species-level eligibility and name mappings are reported in Tables S1–S2.

For the nationwide standardized encounter-richness product, species-level encounter probabilities were generated using the hierarchical multispecies occurrence model described in Section S7 and projected across all 181,054 potential waterbird-habitat cells under standardized checklist conditions. Predictions were generated for a 60-min, 1-km checklist conducted by two observers with average observer experience, and were averaged across the four winter months. Species-specific encounter probabilities were then summed across the 296-species regional pool to obtain standardized expected encounter richness. This metric was used only to describe broad nationwide spatial patterns and should not be interpreted as observed species richness, occupancy or abundance.

S2. Land cover, spatial supports and focal gradients

Annual land-cover proportions were derived from the China Land Cover Dataset for 2015–2024, reprojected from Albers coordinates to WGS84 and aggregated to 0.05° grid cells. The source classes were cropland, forest, shrubland, grassland, water, snow/ice, barren land, impervious surface and wetland. Period P1 used means for 2015–2019 and P2 used means for 2020–2024. Boundary cells were clipped to the national study domain and retained proportions were renormalized. Potential waterbird habitat comprised cells with water plus wetland cover greater than zero in at least one year, yielding 181,054 candidate cells.

Assemblage models used prespecified gradients for aquatic-resource availability, open-water cover, cropland, impervious cover and landscape-edge structure. Continuous predictors were standardized. At the species level, three focal compositional log-ratio contrasts represented aquatic resources, impervious cover and agricultural landscapes. A small positive replacement constant was used only to permit logarithms in zero-containing compositions, and the same predefined definitions were applied consistently across species. Because wetland cover was zero in 99.80% of checklist units relevant to the agricultural contrast, this predictor is interpreted as an agricultural-landscape gradient rather than direct wetland-to-cropland conversion.

S3. Functional-trait compilation and seven-dimensional functional space

Morphological traits were obtained primarily from AVONET, life-history traits from the Amniote Database, and diet composition from EltonTraits 1.0. After taxonomy harmonization, missing retained values were imputed with

missForest. Candidate variables were screened for redundancy and multicollinearity before axis construction. A body-size axis was calculated as the first principal component of log body mass, log body length and log wing length. Two beak axes were calculated from log bill length, width and depth. Diet composition was summarized by its first principal component. The seven-dimensional functional space contained Size_PC1, Beak_PC1, Beak_PC2, HWI, log tarsus length, log clutch size and Diet_PC1, each standardized across the 296-species pool.

Under the retained orientations, higher Size_PC1 indicates generally smaller-bodied species; Beak_PC1 mainly captures overall bill size; and Beak_PC2 contrasts relatively long, narrow bills with shorter, broader or deeper bills. Tables S3–S6 provide the species-level values, PCA loadings, variance explained and explicit sign semantics. Figure S2 reports correlations among the seven retained axes. The same matrix was used to calculate functional distances and to analyse trait structure in species-specific land-use coefficients.

S4. Coverage-standardized taxonomic and functional diversity

Taxonomic diversity (TD) and functional diversity (FD) were estimated with iNEXT.3D v1.0.12 from raw incidence data using coverage-based rarefaction and extrapolation. The primary coverage target was 0.80; coverage 0.90 and Hill order q = 1 were retained as sensitivity analyses, whereas the prespecified primary results use q = 0. Spatial units required at least 10 checklists, at least three winter months, at least three winter years and at least five detected target species. Extrapolation to the target coverage was additionally constrained such that the number of incidence sampling units required to reach the target coverage did not exceed twice the observed number of sampling units ($m_T \leq 2T$); coverage thresholds were not relaxed for undersampled cells. The final analysis comprised 456 spatial units, including 379 units at 0.05° resolution and 77 units at 0.10° resolution.

TD was coverage-standardized species richness. FD used Euclidean pairwise distances in the standardized seven-axis trait space, divided by the maximum distance in the complete 296-species pool, and the iNEXT.3D area-under-the-curve definition with 50 cut points. Estimates were attempted first at 0.05° resolution; predefined 0.10° supports were used when the finer cell did not pass the sampling gate. Bootstrap uncertainty in the diversity estimates was propagated through 200 response draws in the assemblage models (Table S7).

S5. Assemblage-level land-use models and spatial validation

TD and FD were analysed separately on the log scale using generalized additive models fitted by restricted maximum likelihood with term selection. Each response was compared between a spatial baseline and a full model. The baseline contained the spatial-support indicator and a two-dimensional thin-plate spline of longitude and latitude. The full model added the prespecified land-use terms; the aquatic-resource gradient used a cubic regression spline, whereas open water, cropland, impervious cover and edge entered linearly. Spatial units were weighted inversely to their response uncertainty, with weights normalized to avoid arbitrary changes in effective sample size.

$$\log(D_i) = \beta_0 + \beta_{\text{res}} R_i + s(\text{lon}_i, \text{lat}_i) + s(A_i) + \boldsymbol{\beta}^T \mathbf{L}_i + \varepsilon_i$$

Predictive performance was evaluated with the same five spatial folds for all models. Incremental predictive information was defined as the percentage reduction in out-of-fold RMSE of the full model relative to its spatial baseline. Linear effects were back-transformed to percentage differences in expected diversity for a one-standard-deviation predictor increase. Moran's I with 999 permutations tested remaining residual spatial structure. Tables S8–S10 report coefficients, out-of-fold performance and Moran diagnostics; Figure S3 reports fold stability.

S6. TD–FD synchrony and the trait-label permutation null model

National TD–FD synchrony was described with Spearman rank correlation, Pearson correlation after log transformation and a prespecified nonlinear generalized additive model with a cubic regression spline and k = 5. To test whether strong synchrony was species–trait correspondence, we applied a trait-label permutation null model. In each of 999 replicates, the complete seven-axis trait vector of each species moved as an intact block to another species label. The checklist × species incidence matrix, assemblage membership, occurrence frequencies, trait distribution and covariance, coverage target, functional-diversity definition and spatial units were unchanged. FD

was recalculated for all 456 units in every replicate.

Observed Spearman correlation, log-Pearson correlation and nonlinear deviance explained were compared with their replicate-specific null distributions using finite-sample empirical probabilities with a +1 correction. For $SES_{FD,i}$, functional departure was summarized as:

$$SES_{FD,i} = \frac{FD_{\text{obs},i} - \mu\left(FD_{\text{null},i}\right)}{\sigma\left(FD_{\text{null},i}\right)}$$

Negative SES_{FD} indicates that an observed assemblage contains less functional diversity than expected after preserving its richness, incidence and composition while randomizing which trait vector is attached to which species. A secondary SES_{FD} model reused the primary five spatial folds and land-use definitions. Because land use did not improve spatially out-of-fold prediction and residual spatial autocorrelation persisted, this diagnostic is not interpreted as evidence of a transferable land-use mechanism. Tables S11–S13 and Figures S4–S6 contain the complete null and diagnostic results.

S7. Trait-free hierarchical multispecies occurrence model

Species-specific land-use coefficients were estimated without functional traits using a hierarchical Bernoulli-logit model. The design matrix contained 38 checklist-level predictors: log duration, log traveling distance, log observer count, observer experience, protocol, year, sine and cosine terms for seasonal timing, longitude, latitude, the three focal land-use log-ratios and 25 Gaussian spatial radial-basis functions. Continuous predictors were standardized before fitting. Species coefficients were partially pooled through community-, family-, order- and species-level components.

$$logit(p_{js}) = \alpha_s + \sum_{k=1}^{K} X_{jk}\,\beta_{sk}$$

$$Y_{js} \sim Bernoulli(p_{js})$$

$$\beta_{sk} = \mu_k + u_{\text{family}(s),k} + u_{\text{order}(s),k} + u_{s,k}$$

The model was implemented in PyTorch with weighted Bernoulli cross-entropy, Adam optimization and a batch size of 1,024. Five spatial preflight folds selected a median duration of 25 epochs. Five independently initialized full-data fits then generated the coefficient ensemble. Functional traits were absent from this first stage, so the response in the second-stage trait analysis did not already contain trait information.

S8. Cross-species trait models, uncertainty and species holdout

For each focal gradient, the independently estimated species coefficients were modelled simultaneously against all seven standardized functional axes. This produced 21 prespecified trait × gradient relationships. Uncertainty used 1,000 bootstrap replicates; each replicate selected one of the five occurrence-model seeds, resampled species with replacement and refitted the seven-trait model. Table S14 reports all 21 prespecified trait–land-use relationships.

$$\hat{\beta}_{sg} = \alpha_g + \sum_{m=1}^{7} \gamma_{gm}\,T_{sm} + \varepsilon_{sg}$$

Transferability was evaluated in five species-holdout folds defined before examining land-use coefficients. The 176 direct-data species were stratified by ecological group and wetland specificity into folds of 35, 35, 35, 35 and 36 species. For each fold, the model was fitted to four folds and predicted the fifth. The no-trait baseline was the training-species mean coefficient.

$$\Delta RMSE_g = RMSE_{\text{baseline},g} - RMSE_{\text{trait},g}$$

so positive $\Delta RMSE_g$ indicates improved prediction. Overall uncertainty used 3,000 joint bootstrap replicates that selected an occurrence-model seed and resampled the complete out-of-fold species set. Three gradient-level tests

were corrected by the Benjamini–Hochberg procedure. Tables S15–S16 provide gradient-level holdout decisions and joint-bootstrap uncertainty.

S9. Direct-data selection and trait-space representation

The direct-data tier contained 176 species with at least one winter detection on an eligible complete checklist and valid CLCD support. It did not impose a minimum support threshold. Four detected predominantly marine species without valid local CLCD support were assigned to a marine presence-only transfer tier; 116 additional species were all-zero in the frozen checklist set. We compared the 176 direct-data and 120 model-assisted species across the seven standardized axes using standardized mean differences and 9,999 label permutations with Benjamini–Hochberg correction. We also compared the squared Euclidean distance between group centroids in seven-dimensional space with 9,999 label permutations.

S10. Detection and support sensitivity

For each D1 species, model-standardized detection rates were computed at the observed 25th and 75th percentiles of log-duration and observer experience, changing one covariate at a time while retaining all other checklist covariates at their observed values. Predictions were averaged across checklists and five model seeds. We correlated each land-use coefficient with observed and fitted detection rates, duration and expertise contrasts, winter detection count and occupied-grid count. Spearman intervals used 2,000 species bootstrap replicates, and 18 tests were corrected by Benjamini–Hochberg. Agricultural trait models were additionally repeated under four progressively stricter detection/grid/fold support thresholds.

S11. Species-level equal-block bootstrap

Checklists were assigned to 0.25° latitude–longitude blocks. Each of 1,798 blocks received equal total weight, blocks were sampled with replacement in 200 replicates, and agricultural coefficients were updated by a one-step score/information calculation around the fitted hierarchical estimates while nuisance coefficients remained fixed. The seven-trait regression was then refitted to 123 species with at least 20 detections, 10 occupied grids and detections in three spatial folds. This procedure is a first-order block-bootstrap sensitivity analysis rather than 200 complete hierarchical-model refits.

S12. Regional prioritization screen

Richness-constrained SES_{FD} values were summarized by the predefined macroregions and by province after spatially joining unit centroids to the frozen province boundary layer. Province summaries with fewer than five sampled units were not used for interpretation. These are descriptive prioritization screens and do not test local mechanisms.

Supplementary Results

S1. Assemblage-model robustness

Adding land-use terms to the spatial baseline reduced out-of-fold RMSE by 8.14% for TD and 11.74% for FD. Residual Moran's I was 0.0149 for TD ($p = 0.428$) and 0.0053 for FD ($p = 0.704$), providing no evidence of remaining spatial autocorrelation at the tested neighbourhood definition. The directions of the major open-water, cropland and impervious associations were broadly stable across spatial folds, although fold estimates varied in magnitude (Figure S3; Tables S8–S10).

S2. Synchrony relative to randomized trait identity

Observed TD–FD synchrony was lower than every trait-label null replicate for Spearman correlation, log-Pearson correlation and nonlinear deviance explained (empirical low-tail $p = 0.001$ for each). Across units, mean SES_{FD} was

−2.62, median SES_{FD} was −2.54, and 71.05% of units lay outside their unit-specific 95% null envelopes. Land-use variables did not improve spatially out-of-fold SES_{FD} prediction (−1.83%), and residual Moran's I remained 0.092 ($p < 0.001$). Thus the null analysis supports trait-identity-dependent clustering but not a spatially transferable land-use explanation for that clustering (Figures S4–6; Tables S11–S13).

S3. Trait-free cross-species prediction

The seven-trait model improved cross-species prediction most clearly for the agricultural-landscape gradient (ΔRMSE = 0.0374, 95% bootstrap CI = 0.0059–0.0708, q = 0.028). Improvement was weaker and not FDR-supported for the aquatic-resource gradient (ΔRMSE = 0.0151, 95% CI = −0.0057–0.0362, q = 0.135) or the impervious-cover gradient (ΔRMSE = 0.0046, 95% CI = −0.0132–0.0232, q = 0.316). These results indicate spatially transferable multivariate trait structure for the agricultural gradient, whereas trait-level associations along the other gradients should be interpreted independently of gradient-level cross-species predictive support (Tables S14–S16).

S4. Species-level bias and spatial robustness

Direct-data and model-assisted species differed in overall seven-dimensional trait space (permutation P = 0.0001), primarily along Clutch and Diet_PC1 (both q = 0.0007). Size_PC1, Beak_PC1 and HWI agricultural associations retained positive directions across all support thresholds and the equal-block bootstrap. Agricultural coefficients were not significantly correlated with observed or fitted detection rate, detections or occupied grids after correction; duration and expertise correlations were also not FDR-supported. Aquatic-resource coefficients showed modest detection/support correlations, whereas impervious-cover coefficients did not. Regional SES_{FD} screening identified a high prevalence of below-null units in the Eastern Plains and in several middle–lower Yangtze and Yellow Sea coastal provinces, but these summaries do not identify local causes.

Supplementary Figures

Figure S1. Nationwide standardized expected encounter richness.

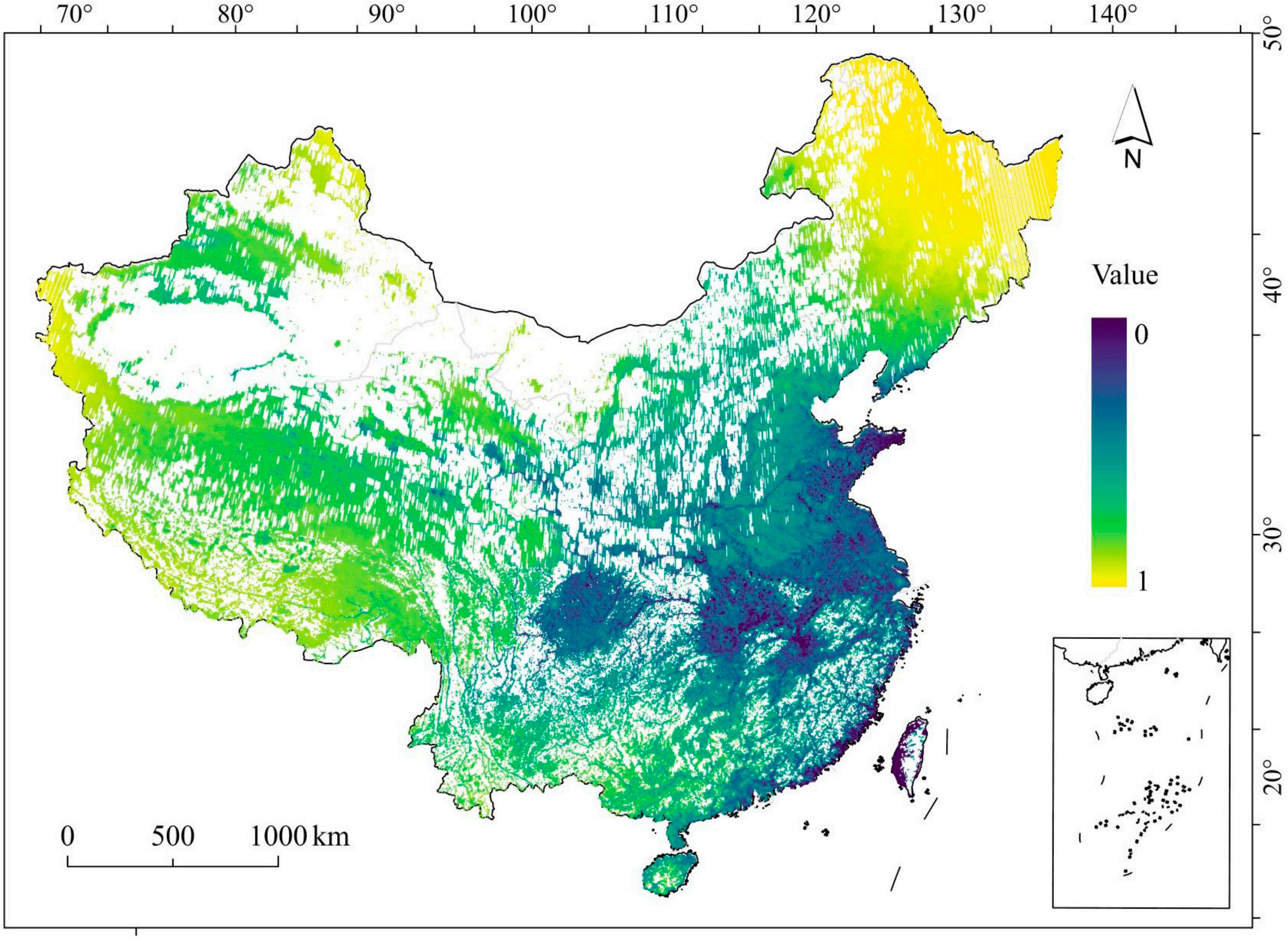

Figure S1. Predicted standardized expected encounter richness across 181,054 potential waterbird-habitat grid cells at 0.05° resolution. Values represent the sum of species-level encounter probabilities predicted under standardized checklist conditions (60 min, 1 km, two observers and average observer experience), averaged across the four winter months. This metric is a standardized spatial index and should not be interpreted as observed species richness, occupancy or abundance.

Figure S2. Correlation among the seven functional-trait axes.

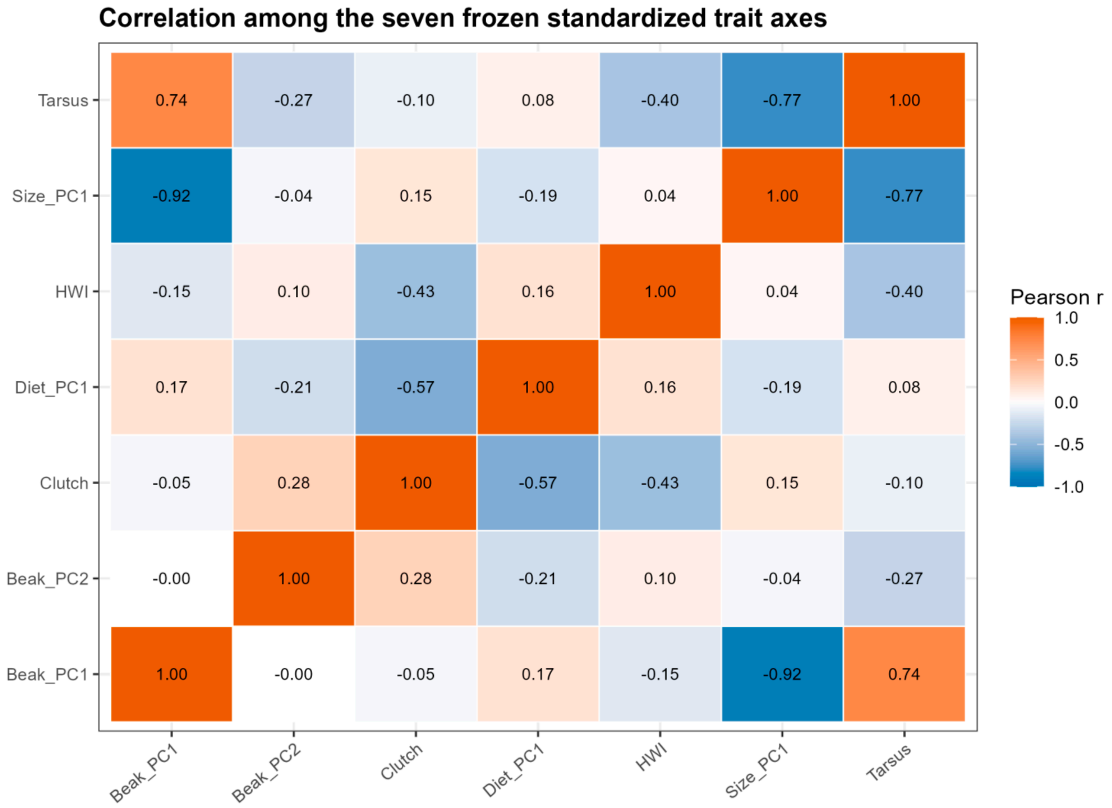

Figure S2. Pearson correlations among the seven standardized axes used to construct functional distances and to analyse cross-species land-use coefficients: Beak_PC1, Beak_PC2, log clutch size, Diet_PC1, hand-wing index (HWI), Size_PC1, and log tarsus length. The matrix is reported to make the remaining dependence among retained axes explicit; all seven axes were nevertheless retained because they represent distinct prespecified functional dimensions.

Figure S3. Five-fold direction stability of assemblage-level land-use associations.

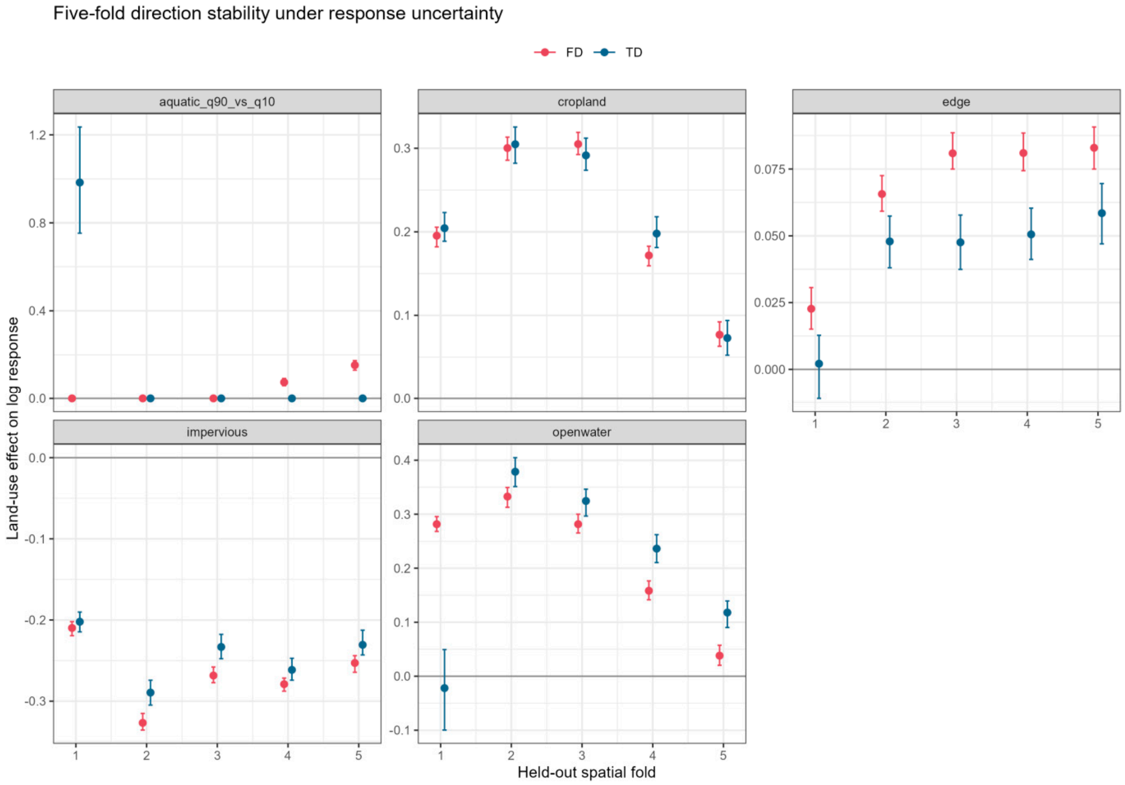

Figure S3. Fold-specific effects on the log response for taxonomic diversity (TD) and functional diversity (FD), with uncertainty propagated from the diversity estimates. Panels show the nonlinear aquatic-resource contrast, cropland, landscape edge, impervious cover, and open-water cover. The figure is a stability diagnostic: fold estimates are not independent inferential tests, and apparent between-fold differences should not be interpreted as regional causal effects.

Figure S4. Trait-label permutation null distribution for log-scale Pearson correlation.

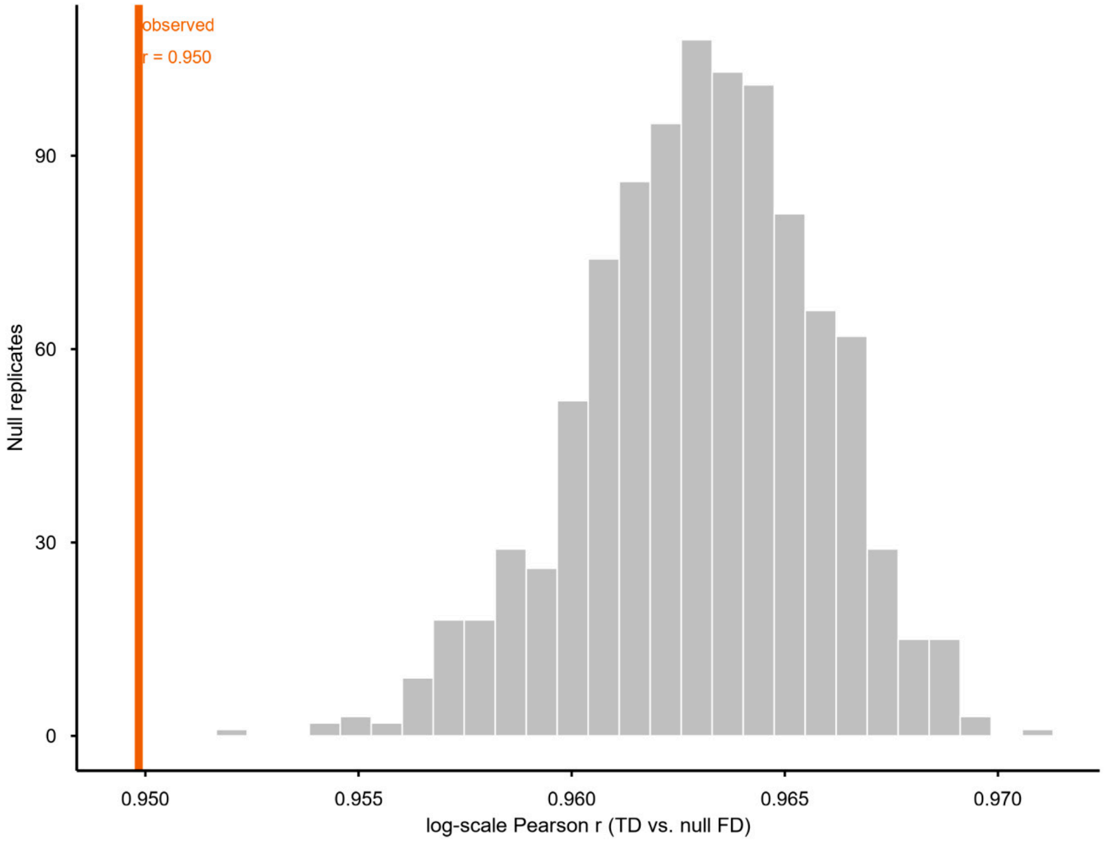

Figure S4. Null distribution of the Pearson correlation between log taxonomic diversity and log functional diversity across 999 permutations. Each permutation reassigned complete seven-dimensional trait vectors among species while preserving checklist incidence, species composition, species occurrence frequencies, and the coverage-standardization procedure. The observed correlation ($r = 0.9498$) was lower than all null values (null mean = 0.9630; 95% null range = 0.9570–0.9680; empirical low-tail $p = 0.001$).

Figure S5. Trait-label permutation null distribution for nonlinear deviance explained.

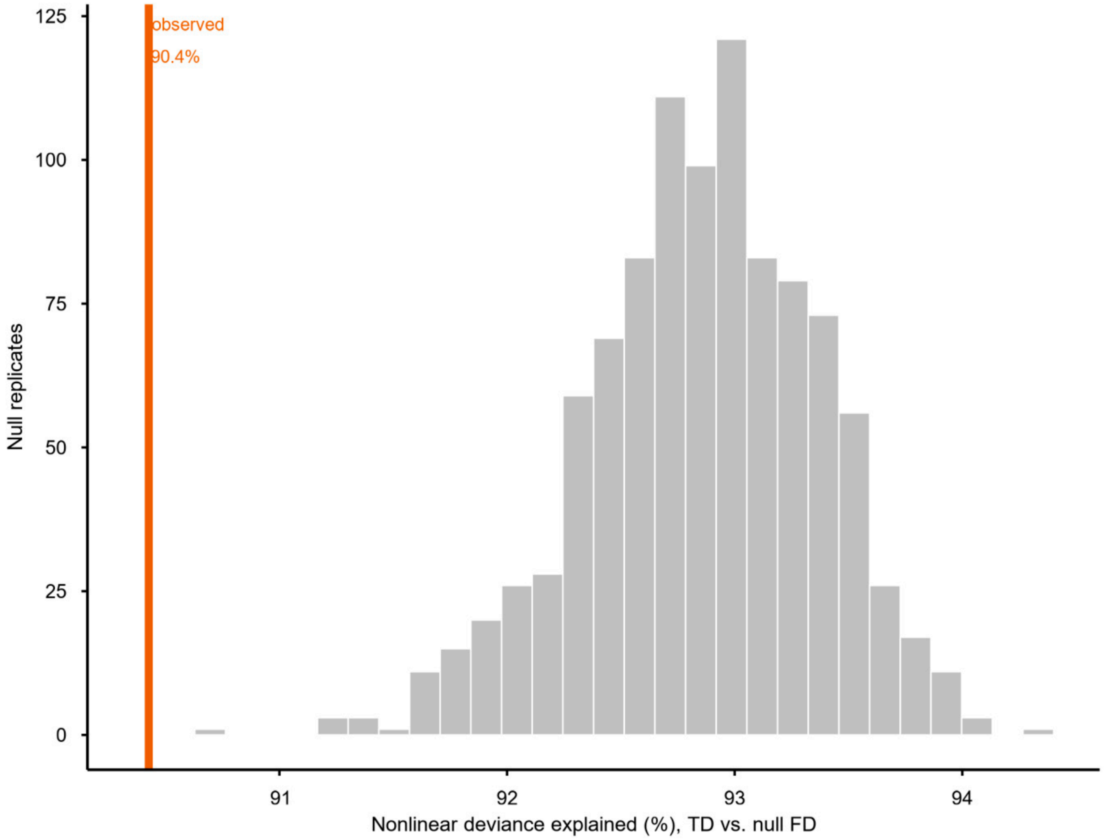

Figure S5. Null distribution of the deviance in functional diversity explained by the prespecified nonlinear taxonomic-diversity model across 999 trait-label permutations. The observed value (90.43%) was lower than all null values (null mean = 92.85%; 95% null range = 91.76–93.79%; empirical low-tail p = 0.001).

Figure S6. Spatial distribution of unit-level standardized functional departure.

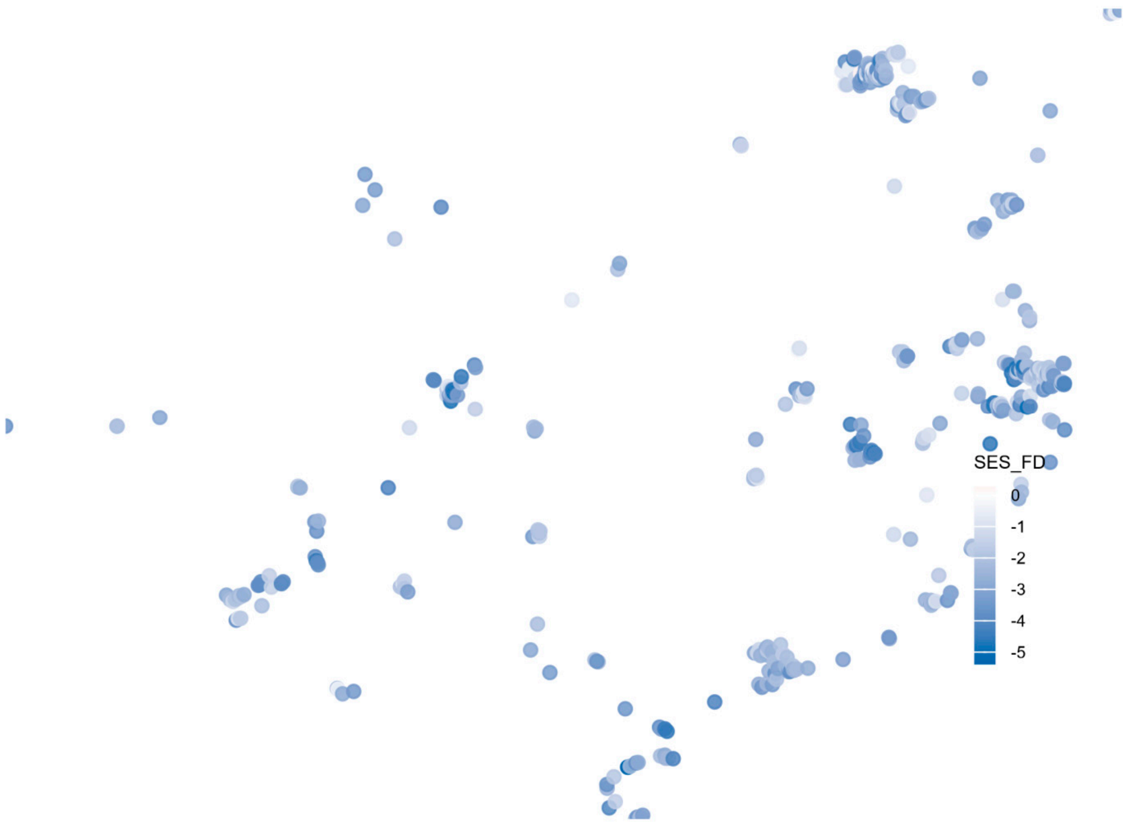

Figure S6. Map of SES_{FD} for the 456 coverage-standardized spatial units, calculated as the difference between observed FD and the unit-specific trait-label null mean divided by the unit-specific null standard deviation. Negative values indicate lower observed functional diversity than expected after preserving the observed assemblage and incidence structure but randomizing species–trait identity. This map is descriptive and does not establish a land-use mechanism.

Figure S7. Direct-data and model-assisted species in the first two principal components of the standardized seven-axis trait space.

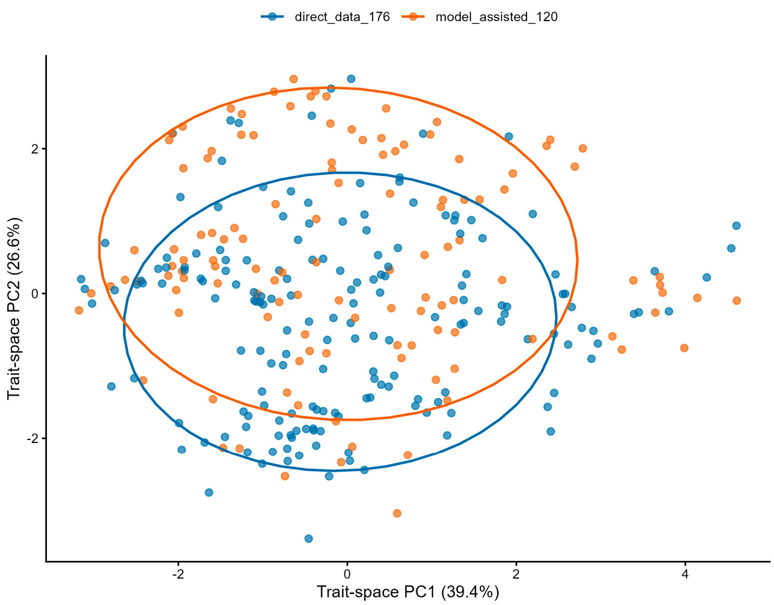

Figure S7. Direct-data (n = 176) and model-assisted (n = 120) species in the first two principal components of the standardized seven-axis trait space. Ellipses show 80% data ellipses and are descriptive; the formal seven-dimensional centroid permutation test is reported in Table S20.

Figure S8. Agricultural-gradient trait associations after equalizing 0.25° spatial-block influence.

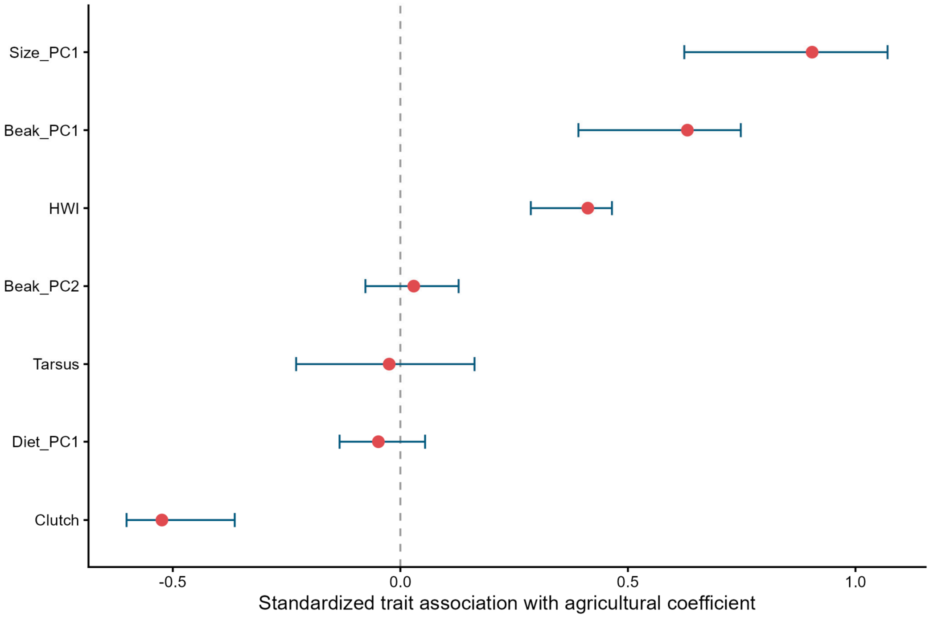

Figure S8. Agricultural-gradient trait associations after equalizing 0.25° spatial-block influence. Points show equal-block one-step estimates and lines show 95% intervals from 200 spatial block-bootstrap replicates for 123 better-supported species.

Methodological References

Aitchison, J., 1986. The Statistical Analysis of Compositional Data. Chapman & Hall, London.

Benjamini, Y., Hochberg, Y., 1995. Controlling the false discovery rate: A practical and powerful approach to multiple testing. J. R. Stat. Soc. Ser. B Methodol. 57, 289–300.

Chao, A., Henderson, P.A., Chiu, C.-H., Moyes, F., Hu, K.-H., Dornelas, M., Magurran, A.E., 2021. Measuring temporal change in alpha diversity: A framework integrating taxonomic, phylogenetic and functional diversity and the iNEXT.3D standardization. Methods Ecol. Evol. 12, 1926–1940.

Dormann, C.F., Elith, J., Bacher, S., Buchmann, C., Carl, G., Carré, G., Marquéz, J.R.G., Gruber, B., Lafourcade, B., Leitão, P.J., Münkemüller, T., McClean, C., Osborne, P.E., Reineking, B., Schröder, B., Skidmore, A.K., Zurell, D., Lautenbach, S., 2013. Collinearity: A review of methods to deal with it and a simulation study evaluating their performance. Ecography 36, 27–46.

Johnston, A., Hochachka, W.M., Strimas-Mackey, M.E., Ruiz Gutierrez, V., Robinson, O.J., Miller, E.T., Auer, T., Kelling, S.T., Fink, D., 2021. Analytical guidelines to increase the value of community science data: An example using eBird data to estimate species distributions. Divers. Distrib. 27, 1265–1277.

Kingma, D.P., Ba, J., 2015. Adam: A Method for Stochastic Optimization. arXiv 2014, arXiv:14126980

Liu, J., Que, P.J., Zhang, Z.W., 2019. Species diversity and suggestions for adjustment of the national protection level of waterbirds in China. Wetland Sci. 17, 123–136.

Moran, P.A.P., 1950. Notes on continuous stochastic phenomena. Biometrika 37, 17–23.

Myhrvold, N.P., Baldridge, E., Chan, B., Sivam, D., Freeman, D.L., Ernest, S.K.M., 2015. An amniote life-history database to perform comparative analyses with birds, mammals, and reptiles. Ecology 96, 3109.

Paszke, A., Gross, S., Massa, F., Lerer, A., Bradbury, J., Chanan, G., Killeen, T., Lin, Z., Gimelshein, N., Antiga, L., Desmaison, A., Köpf, A., Yang, E., DeVito, Z., Raison, M., Tejani, A., Chilamkurthy, S., Steiner, B., Fang, L., Bai, J., Chintala, S., 2019. PyTorch: An imperative style, high-performance deep learning library. Adv. Neural Inf. Process. Syst. 32.

Roberts, D.R., Bahn, V., Ciuti, S., Boyce, M.S., Elith, J., Guillera-Arroita, G., Hauenstein, S., Lahoz-Monfort, J.J., Schröder, B., Thuiller, W., Warton, D.I., Wintle, B.A., Hartig, F., Dormann, C.F., 2017. Cross-validation strategies for data with temporal, spatial, hierarchical, or phylogenetic structure. Ecography 40, 913–929.

Stekhoven, D.J., Bühlmann, P., 2012. missForest—non-parametric missing value imputation for mixed-type data. Bioinformatics 28, 112–118.

Tobias, J.A., Sheard, C., Pigot, A.L., Devenish, A.J.M., Yang, J., Sayol, F., Neate-Clegg, M.H.C., Alioravainen, N., Weeks, T.L., Barber, R.A., Walkden, P.A., MacGregor, H.E.A., Jones, S.E.I., Vincent, C., Phillips, A.G., Marples, N.M., Montaño-Centellas, F.A., Leandro-Silva, V., Claramunt, S., Darski, B., Freeman, B.G., Bregman, T.P., Cooney, C.R., Hughes, E.C., Capp, E.J.R., Varley, Z.K., Friedman, N.R., Korntheuer, H., Corrales-Vargas, A., Trisos, C.H., Weeks, B.C., Hanz, D.M., Töpfer, T., Bravo, G.A., Remeš, V., Nowak, L., Carneiro, L.S., Moncada R., A.J., Matysioková, B., Baldassarre, D.T., Martínez-Salinas, A., Wolfe, J.D., Chapman, P.M., Daly, B.G., Sorensen, M.C., Neu, A., Ford, M.A., Mayhew, R.J., Silveira, L.F., Kelly, D.J., Annorbah, N.N.D., Pollock, H.S., Grabowska-Zhang, A.M., McEntee, J.P., Gonzalez, J.C.T., Meneses, C.G., Muñoz, M.C., Powell, L.L., Jamie, G.A., Matthews, T.J., Johnson, O., Brito, G.R.R., Zyskowski, K., Crates, R., Harvey, M.G., Jurado Zevallos, M., Hosner, P.A., Bradfer-Lawrence, T., Maley, J.M., Stiles, F.G., Lima, H.S., Provost, K.L., Chibesa, M., Mashao, M., Howard, J.T., Mlamba, E., Chua, M.A.H., Li, B., Gómez, M.I., García, N.C., Päckert, M., Fuchs, J., Ali, J.R., Derryberry, E.P., Carlson, M.L., Urriza, R.C., Brzeski, K.E., Prawiradilaga, D.M., Rayner, M.J., Miller, E.T., Bowie, R.C.K., Lafontaine, R.-M., Scofield, R.P., Lou, Y., Somarathna, L., Lepage, D., Iliff, M., Neuschulz, E.L., Templin, M., Dehling, D.M., Cooper, J.C., Pauwels, O.S.G., Analuddin, K., Fjeldså, J., Seddon, N., Sweet, P.R., DeClerck, F.A.J.,

Naka, L.N., Brawn, J.D., Aleixo, A., Böhning-Gaese, K., Rahbek, C., Fritz, S.A., Thomas, G.H., Schleuning, M., 2022. AVONET: Morphological, ecological and geographical data for all birds. Ecol. Lett. 25, 581–597.

Wilman, H., Belmaker, J., Simpson, J., de la Rosa, C., Rivadeneira, M.M., Jetz, W., 2014. EltonTraits 1.0: Species-level foraging attributes of the world's birds and mammals. Ecology 95, 2027.

Wood, S.N., 2017. Generalized Additive Models: An Introduction with R, 2nd ed. CRC Press, Boca Raton, FL.

Yang, J., Huang, X., 2025. The 30 m annual land cover datasets and its dynamics in China from 1985 to 2024, Version 1.0.4 [dataset]. Zenodo. https://doi.org/10.5281/zenodo.15853565
